# Xylem cell size regulation is a key adaptive response to water deficit in *Eucalyptus grandis*

**DOI:** 10.1093/treephys/tpae068

**Published:** 2024-06-18

**Authors:** Rafael Keret, David M Drew, Paul N Hills

**Affiliations:** Institute for Plant Biotechnology, Department of Genetics, Stellenbosch University, Private Bag X1, Matieland, Stellenbosch 7602, South Africa; Department of Forestry and Wood Sciences, Stellenbosch University, Bosman St, Stellenbosch 7599, South Africa; Department of Forestry and Wood Sciences, Stellenbosch University, Bosman St, Stellenbosch 7599, South Africa; Institute for Plant Biotechnology, Department of Genetics, Stellenbosch University, Private Bag X1, Matieland, Stellenbosch 7602, South Africa

**Keywords:** embolism, tracheary elements, transcriptomics, wood anatomy, xylogenesis

## Abstract

Future climatic scenarios forecast increasingly frequent droughts that will pose substantial consequences on tree mortality. In light of this, drought-tolerant eucalypts have been propagated; however, the severity of these conditions will invoke adaptive responses, impacting the commercially valuable wood properties. To determine what mechanisms govern the wood anatomical adaptive response, highly controlled drought experiments were conducted in *Eucalyptus grandis* W. Hill ex Maiden, with the tree physiology and transcriptome closely monitored. In response to water deficit, *E. grandis* displays an isohydric stomatal response to conserve water and enable stem growth to continue, albeit at a reduced rate. Maintaining gaseous exchange is likely a critical short-term response that drives the formation of hydraulically safer xylem. For instance, the development of significantly smaller fibers and vessels was found to increase cellular density, thereby promoting drought tolerance through improved functional redundancy, as well as implosion and cavitation resistance. The transcriptome was explored to identify the molecular mechanisms responsible for controlling xylem cell size during prolonged water deficit. Downregulation of genes associated with cell wall remodeling and the biosynthesis of cellulose, hemicellulose and pectin appeared to coincide with a reduction in cellular enlargement during drought. Furthermore, transcript levels of NAC and MYB transcription factors, vital for cell wall component biosynthesis, were reduced, while those linked to lignification increased. The upregulation of *EgCAD* and various peroxidases under water deficit did not correlate with an increased lignin composition. However, with the elevated cellular density, a higher lignin content per xylem cross-sectional area was observed, potentially enhancing hydraulic safety. These results support the requirement for higher density, drought-adapted wood as a long-term adaptive response in *E. grandis*, which is largely influenced by the isohydric stomatal response coupled with cellular expansion-related molecular processes.

## Introduction

The process of wood formation in trees leads to the production of one of the most valuable global commodities. Wood provides a renewable feedstock for timber, cellulosic products and energy from second-generation biofuels. In addition to these tangible commodities, trees sequester carbon from the atmosphere and play an important role in regulating climate. However, the narrative surrounding climate change tends toward an increase in global temperatures accompanied by a reduction in rainfall (Zwieniecki and Secchi 2015; Schumann et al. 2019).

This unavoidable global issue has accelerated the propagation of various *Eucalyptus* species, some of which are regarded as highly drought-tolerant. Of these, *Eucalyptus grandis* W. Hill ex Maiden is among the most important for timber and pulpwood industries (Ployet et al. 2018). Although highly adaptable, eucalypts can succumb to severe drought stress if the balance between carbon starvation and hydraulic failure cannot be maintained. Initially, trees can respond to drought via stomatal closure, to reduce water tension in the xylem and thus decrease the probability of embolism-induced hydraulic failure (Schumann et al. 2019; Saunders and Drew 2022). However, this is a short-term adaptive strategy, as prolonged stomatal closure will reduce the CO_2_ uptake necessary for photosynthesis, thus resulting in carbon starvation (Qaderi et al. 2019). Ultimately, an important long-term survival strategy involves modifications to the wood’s anatomical structure to mitigate the probability of hydraulic failure (Rodriguez-Zaccaro and Groover 2019).

Wood formation follows a series of highly coordinated biological processes that originate from the lateral meristem known as the cambium (Zhu et al. 2023). Thereafter, cambial derivatives differentiate via cellular expansion, secondary cell wall (SCW) deposition and programmed cell death to form tracheary elements or fibers (Keret et al. 2023). These cells can expand or elongate both symplastically and intrusively (Miodek et al. 2021). The turgor pressure originating from the vacuole provides the driving force, while cellulose microfibril orientation, under the control of cortical microtubules, depicts the direction of cellular expansion (Funada et al. 2016). Furthermore, control of expansion is governed by the extensibility of the cell wall, which is influenced by the remodeling of cellulose, hemicellulose and pectin (Qiu et al. 2021). Cellulose microfibrils are tightly tethered by xyloglucan or xylan–pectin linkages to create an interconnected network that forms the primary load-bearing components of the cell wall (Cosgrove 2016; Saffer 2018). Degradation of xyloglucan or pectin linkages, within these networks, is suggested to enable primary cell wall expansion (Haas et al. 2021). Upon cellular maturity, the SCW is formed and consists primarily of cellulose, hemicellulose and lignin (Zhong et al. 2019). The synthesis, modification and incorporation of these components into the SCW are a crucial determinant of the wood’s physiochemical properties, structure, function and responses to drought.

Water stress can lead to major changes in xylem anatomy, of which the density and size of cells regularly seem to be the most striking feature (Pfautsch et al. 2016; Tng et al. 2018; Wildhagen et al. 2018; Yu et al. 2021). Often, smaller tracheary elements are regarded as hydraulically safer, offering increased implosion resistance, a higher surface area to volume ratio and less pitted wall area to mitigate air-seeding (Badel et al. 2015; Jacobsen et al. 2019; Jacobsen and Pratt 2023). Pit membranes are also affected by the thickness of the conduit walls, which improve the structural integrity of these structures and prevent stretching (He et al. 2023). Moreover, to avoid cavitation-induced hydraulic failure, an increased vessel frequency provides a functionally redundant safety mechanism to ensure that a higher number of small vessels remain functional at any given time (Zwieniecki and Secchi 2015; Islam et al. 2018). Decreased fiber lumen area has also been shown to correlate with an increased cavitation resistance since the mechanical properties of fibers can prevent the formation of microcracks, vessel pit membrane deflection or any other stresses that can encourage vessel collapse (Jacobsen et al. 2005). Despite the clear importance of these functional properties of the xylem and a fast-growing body of research in the area of molecular biology, the processes governing xylem expansion in response to drought remain poorly characterized and require attention in future research (Rodriguez-Zaccaro and Groover 2019; Zhang et al. 2021).

Over the past decade, transcriptomics has emerged as the mainstream approach for disentangling the molecular processes associated with wood formation and the stress response in trees (Piot et al. 2022). For instance, the differential expression of SCW biosynthetic genes and transcriptional regulation of abscisic acid were proposed to correlate with the formation of smaller, high-density vessels and fibers with thicker cell walls in drought stressed poplar (Yu et al. 2021). Interestingly, Yu et al. (2021) reported unexpected patterns of gene expression in the transcriptome under drought, with certain SCW biosynthetic genes and transcription factors (TFs) showing downregulation, whereas others involved in cell wall modifications and cellulose biosynthesis were upregulated. These findings seem to suggest the presence of completely distinct wood formation modules or gene sets, each of which are independently activated to form either normal or ‘stress wood’ (i.e. under abiotic stress). Recently, RNAseq analysis of the *E. grandis* stem transcriptome has revealed that genes associated with growth and metabolism were downregulated during drought, while responses to reactive oxygen species (ROS) and phytohormone signaling pathways were upregulated (Teshome et al. 2023). However, since the xylem anatomy was not considered, no parallels between the gene expression and adaptive wood structure could be drawn. Similarly, studies by Ployet et al. (2019) demonstrated that water exclusion mainly enriched gene ontologies associated with stress, including responses to ROS, chemical stimulants and abscisic acid. It was found that potassium fertilization and not water deficit, enriched most of the gene ontologies associated with cell wall biosynthesis, primary metabolism and cellular development, manifesting in significant changes in the vessel and wood properties (Ployet et al. 2019).

Although studies have identified that processes such as the cell cycle, cell wall organization or biogenesis, cellulose, hemicellulose and lignin biosynthesis are differentially regulated under drought, many have failed to include detailed quantitative wood anatomy metrics that may offer further insights into how these genes influence the drought adaptive phenotype (Tang et al. 2015; Wildhagen et al. 2018; Coleman et al. 2021; Teshome et al. 2023). To assess whether expansion-related genes can be linked to tree physiology and wood properties, a transcriptomic study was conducted on *E. grandis* subject to controlled drought exposure. Given that the processes governing xylem cell expansion, cellulose and hemicellulose synthesis display diurnal plasticity, primarily occurring predawn/night (Solomon et al. 2010; Sato et al. 2016; Buttò et al. 2021), we focused on the predawn transcriptome. We hypothesized that a concert of molecular role players that govern cellular expansion and component biosynthesis is set in motion to promote the development of hydraulically safer xylem in *E. grandis* during drought. The identification of these molecular role players is key to understanding how eucalypts and potentially other trees, can adapt their wood properties to cope with future climate scenarios and can be a target for tree improvement or selection programs.

## Materials and methods

### Plant material establishment and watering regime selection

Rooted *E. grandis* ramets were received from Hans Merensky holdings and repotted into 1.5 L bags containing a soil mixture of 1:1 (V/V) palm peat to filter sand with 2 g of Osmocote Pro controlled-release fertilizer (Gauteng, South Africa; Osmocote). The cuttings were grown for 6 months in a nursery (33°55′35.6″S, 18°52′03.3″E) until a height of 45 to 50 cm was achieved. A pilot study was conducted with four watering regimes to determine what amount of water would induce a sufficient drought response without entirely preventing secondary growth. Numerous physiological parameters were considered, including the pre-dawn leaf water potential (PDLWP), stomatal conductance (*g_sw_*), transpiration and stem diameter (SD) that served as proxies for the amount of drought pressure applied to the plants. Considering these physiological parameters, it was deduced that 180 mL of water daily was sufficient for the controls, while 60 mL was shown to elicit a suitable drought response (see Method S1 available as Supplementary data at *Tree Physiology* Online).

### Growth conditions and treatments

Twenty-four saplings were transferred from the nursery into a growth chamber. These plants were randomly assigned into control or drought treatment groups (*n* = 12), positioned according to a randomized Latin square design and acclimated for a period of 7 days receiving 180 mL of water daily. Subsequently, the experimentation commenced by providing the controls with 180 mL of water daily, while the droughted plants received a daily supply of 60 mL, over a period of 30 days. Water content reflectometers (CS655-L, Campbell Scientific, Cape Town, South Africa) were used to record the soil volumetric water content. Maxim I-buttons (DS1922S, Fairbridge Technologies, Sandton, South Africa) recorded a mean ambient temperature of 25 ± 1 °C and a humidity of 75 ± 5.66% throughout the experiment. Using 900 mm Nano LED grow battens (T8, The Lamphouse, Johannesburg, South Africa), the photoperiod was set to 16 h, with a dark period of 8 h. Pyranometers (CS300L, Campbell Scientific) coupled to a CR1000× datalogger (Campbell Scientific) measured an average photosynthetically active radiation of 50 ± 1 μmol photons m^2^ s^−1^ in the middle of the canopy and 111 ± 0.8 μmol photons m^2^ s^−1^ at three quarters canopy level. This experiment was replicated three times independently (*n* = 36).

### Physiological measurements and material harvest

At the start of the experiment, reference markings were made on the stem of each plant, exactly 30 cm below the apical bud. This marking served as a reference to accurately measure the SD every third day using digital vernier calipers (GV9371, Builders Express, Stellenbosch, South Africa), as well as the relative change in height over 30 days (see Tables S1 and S2 available as Supplementary data at *Tree Physiology* Online). The *g_sw_* value was measured every second day, 1 h into the light period, on the first three fully expanded leaves using a LI-COR LI-600 porometer/fluorometer (Campbell Scientific; see Table S3 available as Supplementary data at *Tree Physiology* Online). After the 30-day growth experiment, stem scrapings from the outermost xylem material were collected 31 to 40 cm below the apical bud. These were pooled (i.e. three plants per pool) to produce eight samples per treatment (*n* = 8; see Table S4 available as Supplementary data at *Tree Physiology* Online). From these samples, the lignin composition and SG ratio were quantified at SAPPI Southern Africa Technology Centre (Pretoria, South Africa), using the Klasons lignin and thioacidolysis methods (Schwanninger and Hinterstoisser 2002; Robinson and Mansfield 2009). All abiotic and physiological data were analyzed using the R System for Statistical Computing, Version 4.3.1 (R Core Team 2022; see Code S1 available as Supplementary data at *Tree Physiology* Online).

### Histology and QuPath bioimage analysis

The histology and microscopy protocols applied in the present study are outlined in detail in Keret et al. (2024). In brief, stem segments were harvested from all plants, 30 to 31 cm below the apical bud (*n* = 36) and embedded in paraffin wax (411,663; Merck, Darmstadt, Hesse, Germany). The samples were transversely sectioned at 6 μM, transferred to microscope slides and stained with Safranin-Alcian blue (84,120 & A5268; Merck, Darmstadt, Hesse, Germany). These slides were scanned under a 20× objective with a Nikon eclipse Ni-E upright motorized microscope (Nikon Corporation, Düsseldorf, North Rhine-Westphalia, Germany) coupled to a Nikon DS-Fi2 camera.

The scanned microsection images were analyzed using QuPath bioimage analysis software v0.4.4, as described by Keret et al. (2024). A circular region of interest (0.146 mm^2^) was created within 0.5 mm of the cambium to analyse the wood formed over the experimental period. Quantitative wood anatomy data for both watering conditions were obtained for xylem fibers and vessels (see Tables S5–S7 available as Supplementary data at *Tree Physiology* Online). Characteristics such as the cell area (CA), lumen area (LA) and lumen-to-cell area ratio (L/C) were directly measured, whereas the cell wall area (CWA), cell wall thickness (CWT), density, fractional cell wall area, fractional lignin area, vessel implosion resistance and theoretical hydraulic conductivity were derived from QuPath variables (Scholz et al. 2013; Pfautsch et al. 2016; Yu et al. 2021; see Code S2 available as Supplementary data at *Tree Physiology* Online).

### RNA extraction

Two hours before the start of the photoperiod, the bark and phloem were removed and the developing xylem (including cambium) was harvested using the scraping technique, to enrich for mostly xylem tissue (Ployet et al. 2019; Zhu et al. 2023). The stem scrapings were collected directly after the 30-day experiment, 25 to 30 cm below the apical bud and immediately flash-frozen in liquid nitrogen. Four pooled RNA samples were generated for both the control and droughted conditions, each containing material from three plants (*n* = 4). Total RNA was extracted using a modified CTAB extraction protocol (White et al. 2008; see Method S2 available as Supplementary data at *Tree Physiology* Online). The RNA was assessed for integrity and quantity at the Stellenbosch University Central Analytical Facilities (CAF, Stellenbosch, South Africa), using the RNA ScreenTape system and QuBit assay, respectively. Subsequently, quality control checks at Macrogen (Macrogen Europe, Amsterdam, The Netherlands) confirmed high-quality RNA, with RIN scores all above nine. Finally, to assess the level of tissue enrichment, xylem, cambium and phloem-specific primers were designed in OligoExplorer v1.1.2 for RT-PCR amplification (https://oligo-explorer.software.informer.com/1.2/). According to the manufacturer’s instructions, the RevertAid First Strand cDNA Synthesis Kit (K1621, Thermo Fisher Scientific, Johannesburg, South Africa) was used to synthesize cDNA for RT-PCR amplification. The RT-PCR amplified almost exclusively xylem (Eucgr.J00938.1, *FLAP11*) and cambium (Eucgr.A02902, *PXY*) specific genes, with virtually no amplification of the phloem (Eucgr.I01715.1, *DP3*) specific gene. Positive controls were used to confirm the expression of all target genes in the stem tissues of *E. grandis* (see Method S3 available as Supplementary data at *Tree Physiology* Online).

### Transcriptomic analysis and annotation

Next-generation RNA Sequencing (RNA-Seq) was performed by Macrogen (NCBI; PRJNA1012834). In summary, the TruSeq Stranded mRNA LT Sample Prep Kit (20020595, Illumina, Europe) was used to prepare eight cDNA libraries (four replicates each of control and droughted samples) that were sequenced using the Novaseq 6000 Illumina platform, to generate 20 million paired end reads per sample. Trimmomatic v0.3.2 was used to remove base calls displaying a Phred quality score below 20, trim Illumina adapters (ILLUMIMACLIP: TruSeq3-PE: 2:30:10) and omit reads with fewer than 36 base pairs (Bolger et al. 2014). The RNA sequencing libraries were inspected in FastQC v0.11.9 before and after trimming (Andrews 2010). Remaining high-quality reads were mapped to the *E. grandis* reference genome (https://www.ncbi.nlm.nih.gov/datasets/genome/GCF_000612305.1/) using HGFM indexes with transcripts, in Hisat2 v2.2.1 (Kim et al. 2019). The mapped reads were assembled into a counts matrix using the Subread FeatureCounts v2.0.5 package (Liao et al. 2014). Genes with five or fewer read counts across all samples were omitted. DESeq2 of Bioconductor was applied in R for library normalization (median of ratios), removal of outliers (Cook’s distance) and significance testing of differentially expressed genes using the default parameters (R Core Team 2022; Love et al. 2014; https://github.com/Rafael-Keret/Eucalyptus_transcriptomic_analysis/tree/main/1.DESeq2).

Annotation of the *E. grandis* genes followed a modified procedure from Zhu et al. (2023). In summary, 31,415 genes from the DESeq2 analysis were exported to extract *E. grandis* gene models (i.e. coding sequences) from the NCBI datasets portal, using the ‘Command line tools’ option. Subsequently, the latest ARAPORT11 genome release (https://www.arabidopsis.org/download_files/Proteins/Araport11_protein_lists/Araport11_pep_20220914.gz) was downloaded from TAIR to create a local protein database. Translated nucleotide BLAST (blastx, *e*-value ≤ 0.001) of the *E. grandis* gene models was conducted against the database to identify the closest orthologs in *Arabidopsis thaliana*. Hereafter, the prefix ‘*Eg*’ is used to denote these putative orthologs in *E. grandis*, whereas no prefix indicates well-characterized genes in other species.

### GSEA and gene functional characterization

Gene Ontology (GO), symbol and functional annotation were assigned to the *E. grandis* ENTREZ IDs using the *A. thaliana* orthologs in combination with clusterProfiler and biomaRt (Smedley et al. 2009; Wu et al. 2021; https://github.com/Rafael-Keret/Eucalyptus_transcriptomic_analysis/tree/main/2.Gene_anotation). To explore the biological themes underpinning the gene expression data, a gene set enrichment analysis (GSEA) was performed using clusterProfilers non-model organism feature with a Benjamini–Hochberg *P*_adj_ cutoff of 0.05 and a gene set size of 50 to 480. An enrichment network map was constructed from these ontologies. Finally, differentially expressed genes displaying absolute log_2_ fold change > 1 and a *P*_adj_ < 0.05 (false discovery rate corrected) were filtered from the dataset for functional exploration using a combination of the biomaRt annotations and the platform MapMan (Thimm et al. 2004). Ontologies and gene sets associated with the stress response, cell cycle, cell wall remodeling and component biosynthesis were mined from the transcriptome with search queries generated in R (https://github.com/Rafael-Keret/Eucalyptus_transcriptomic_analysis/tree/main/3.Functional_characterization_structural). Gene expression candidates with a high relevance to fiber and vessel development (i.e. xylogenesis) were explored and subsequently linked to the anatomical phenotypes observed under each watering regime.

### Statistical analysis

Prior to analysis, all datasets were tested for normality using a combination of the quantile-quantile plot and Shapiro–Wilks test, with residual versus fit plots used to assess the homogeneity of the variance for models (Kozak and Piepho 2018). Data failing any of the above assumptions were either log or square root transformed, followed by analysis with a *t*-test or repeated measures ANOVA (Tukey’s *post hoc* test). Data still failing these assumptions after transformation were analyzed using the Wilcoxon signed-rank test. All statistical analysis, data visualizations and custom figures were constructed using R v4.3.1 software (R core team 2022).

## Results

### Physiological and wood anatomical adaptations to drought

**Figure 1:**
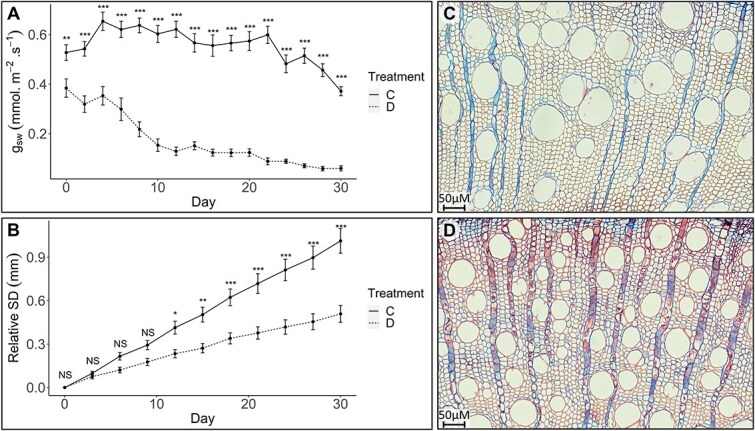
Depiction of the physiological and wood anatomical responses of *Eucalyptus grandis* to drought. (A) Comparison of the stomatal conductance (*g*_sw_) and (B) relative SD over the course of 30 days, between the control and droughted treatments. Transverse microsections of (C) control and (D) droughted wood samples displaying the differences in wood anatomical features elicited by low water conditions. The sections were imaged under a 20× objective lens and stained with Safranin-Alcian blue. Control (C) and droughted (D) treatments have been abbreviated in the figures. Significant and nonsignificant differences are represented as asterisks (^*^P ≤ 0.01, ^*^^*^P ≤ 0.001, ^*^^*^^*^P ≤ 0.0001) or NS, respectively.

To determine the physiological and wood anatomical responses of *E. grandis* to drought, the average daily soil moisture was significantly (*P* < 0.01) reduced from 0.283 ± 0.0012 m^3^ m^−3^ in the controls to 0.065 ±0.0007 m^3^ m^−3^ for the drought treatment. This reduction in water content significantly (*P* < 0.01) reduced the *g_sw_* of the droughted plants compared with controls throughout the experiment. Water deprivation caused the *g_sw_* value to decline sharply from Day 4 before stabilizing at Day 10, thereafter showing a steady to gradual decline (Fig. 1A). Control plants displayed a significantly (*P* < 0.01) greater change in SD from Day 12 (Fig. 1B) and similarly grew taller (Table 1).

**Table 1:** Stem and cell anatomical properties of *Eucalyptus grandis* in response to drought and control conditions. The data represent the mean of measurements taken throughout the study, with the respective standard error indicated in parenthesis. A *t*-test or a Wilcoxon-sign ranked test was implemented to determine significant differences between the treatments for normal and nonnormal data, respectively.

Organ or cell	Property	Control	Drought	*P*-value
Stem	Change in height (cm)	15.25 (0.65)	7.50 (0.39)	˂0.01
	Klasons lignin (%)	30.09 (1.68)	29.10 (2.06)	0.72
	SG ratio	2.84 (0.03)	2.69 (0.06)	0.06
	Fractional lignin area (%)	11.88 (0.12)	12.26 (0.13)	0.04
	Fractional cell wall area (%)	39.49 (0.41)	42.12 (0.47)	0.01
Fiber	Cell area (μm^2^)	91.97 (1.53)	82.93 (1.51)	˂0.01
	Lumen area (μm^2^)	46.6 (1.20)	40.77 (1.18)	˂0.01
	Lumen to cell area ratio	0.47 (0.005)	0.45 (0.005)	0.06
	Cell wall area (μm^2^)	45.36 (0.48)	42.16 (0.44)	˂0.01
	Cell wall thickness (μm)	3.26 (0.02)	3.23 (0.019)	0.32
	Density (cells/mm^-2^)	5443.0 (137.9)	6239.8 (152.6)	˂0.01
Vessel	Cell area (μm^2^)	2844.69 (87.4)	1897.96 (57.1)	˂0.01
	Lumen area (μm^2^)	2512.05 (81.1)	1638.86 (52.8)	˂0.01
	Lumen to cell area ratio	0.87 (0.002)	0.84 (0.003)	˂0.01
	Cell wall area (μm^2^)	332.65 (6.95)	259.10 (5.19)	˂0.01
	Cell wall thickness (μm)	3.78 (0.02)	3.73 (0.02)	0.12
	Density (cells mm^-2^)	63.89 (2.74)	88.24 (5.38)	˂0.01
	Implosion resistance (*t*/*b*)^2^	0.006 (0.0007)	0.011 (0.001)	˂0.01
	Theoretical hydraulic conductance (kg s^−1^ m^−1^ MPa^−1^)	32.49 (1.59)	20.81 (1.41)	˂0.01

There were significant differences in various wood properties between the treatment and control plants (Fig. 1C and D). Most strikingly, the drought invoked reductions in the CA and LA for both xylem vessels and fibers (Table 1). Consequently, a decrease in the L/C from 0.87 to 0.84 (*P* < 0.01) was observed in the vessels of water-stressed plants, whereas the fibers displayed a non-significant reduction from 0.47 to 0.45 (*P* = 0.06). Although there was an increase (*P* < 0.01) in the CWA of fibers (42.16 to 45.36 μm^2^) and vessels (259.10 to 332.65 μm^2^) in control plants, no differences in the CWT were present (Table 1). Under drought conditions, a significant (*P* < 0.01) increase in cellular density from 5443.0 to 6239.8 cell mm^-2^ for fibers and 63.89 to 88.24 cell  mm^-2^ in vessels (*P* = 0.01) increased the fractional cell wall area by 2.63%. Ultimately, these changes in wood properties improved the implosion resistance of vessels produced during drought, but with an associated reduction in theoretical hydraulic conductance (Table 1).

### Overview of the molecular drought response

**Figure 2:**
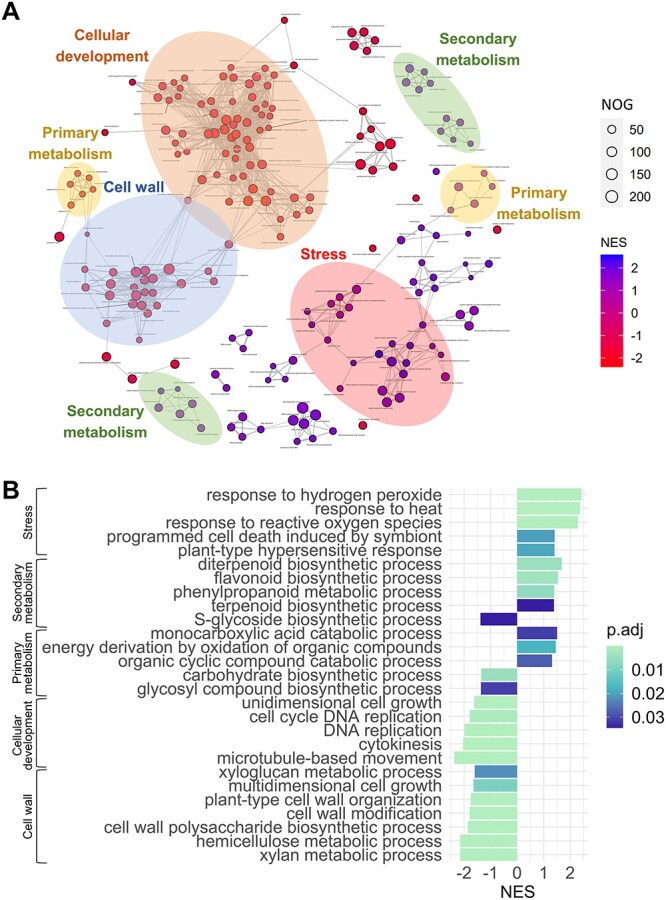
(A) Enrichment network map plot, displaying the enriched or depleted gene ontologies that were triggered in *Eucalyptus grandis* in response to droughted conditions. (B) Histogram displaying the significantly enriched or depleted gene ontologies with putative roles in governing the *E. grandis* stem anatomy during drought. The results represent GSEA using the clusterProfiler 4.0 package in R v4.3.1 software (R Core Team 2022). The adjusted *P*-value (*P*_adj_) is based on the Benjamini–Hochberg correction for multiple comparisons. The figure legends represent the number of genes and normalized enrichment score.

The clear differences in wood anatomy observed in drought versus control plants were further investigated via transcriptomic analysis to determine the changes in gene expression associated with the anatomical phenotypes. Among the 31,414 genes detected in *E. grandis* xylem, 23,368 were successfully assigned an *A. thaliana* ortholog and GO term. The GSEA revealed a total of 202 biological process ontologies that were either significantly (*P*_adj_ < 0.05) enriched or depleted under drought (see Table S8 available as Supplementary data at *Tree Physiology* Online). Assessment of these ontologies revealed many themes occurring within these gene sets. Interestingly, cellular development and cell wall-related ontologies were downregulated in the droughted plants, whereas secondary metabolism and stress-related themes were upregulated (Fig. 2A). In the context of primary metabolism, drought appeared to mainly induce catabolic processes, as opposed to anabolic processes occurring in the controls (Fig. 2B). Upon careful inspection of the gene ontologies, many were identified as putative candidates for regulating the primary and SCW development in *E. grandis*, with the most crucial and nonredundant ontologies outlined in Fig. 2B.

### Genetic drivers of cellular expansion phenotype

**Figure 3:**
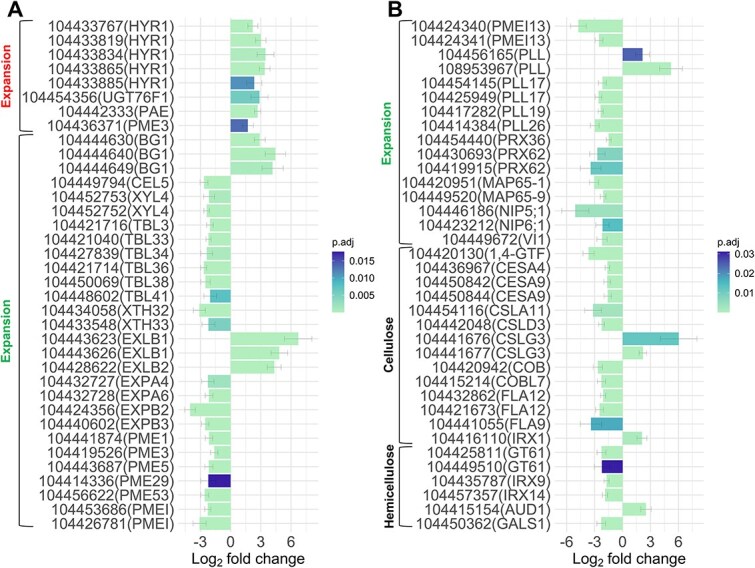
Significantly differentially expressed genes with a putative role in cell size regulation for *Eucalyptus grandis*. The log_2_ fold change was calculated with respect to the droughted versus control condition. The histograms represent gene expression candidates associated with various processes such as (A/B) cellular expansion, (B) cellulose and hemicellulose metabolism. The adjusted *P*-value is based on the Benjamini–Hochberg correction for multiple comparisons.

Once a general overview of the genetic responses in control and droughted plants was obtained, a targeted analysis of the expression data was employed. Overall, 3048 differentially expressed genes were filtered (*P*_adj_ < 0.05, |log_2_ fold change| > 1) from the dataset and all were successfully functionally annotated (see Table S9 available as Supplementary data at *Tree Physiology* Online). As the cell size of fibers and vessels was a clearly contrasting feature of the developing xylem in response to water deficit, genes with direct and supplementary roles in cell size regulation were primarily explored for this analysis (Fig. 3).

Genes linked to the inhibition of cell expansion, such as *hypostatin resistance* (*EgHYR*) *1*, *UDP-glucosyltransferase* (*EgUGT*) *76F1*, *pectin acetylesterase* (*EgPAE*) and *pectin methylesterase* (*EgPME*) *3*, exhibited increased expression during drought. Despite these repressors, some genes that promote cell expansion, such as *beta-1,3-glucanase* (*EgBG*) *1*, *expansin-like* (*EgEXL*) *B1/B2* and *pectin lyase-like* (*EgPLL*), were also elevated in the droughted plants. However, it seems that many genes with putative roles in cellular enlargement were downregulated compared with the controls (Fig. 3A and B). These include *cellulase* (*EgCEL*) *5*, *beta-D-xylosidase* (*EgXYL*) *4*, *trichome birefringence-like* (*EgTBL*) *3/33/34/36/38/41*, *xyloglucan endotransglucosylase/hydrolase* (*EgXTH*) *32/33*, *expansin* (*EgEXP*) *A4/A6/B2/B3*, *peroxidase* (*EgPRX*) *36/62* and *microtubule-associated protein* (*EgMAP*) *65–1*/*65–9* which are described as key players in cell wall loosening and remodeling. Additionally, certain genes potentially influencing elongation, such as *EgPME1/3/5/29/53*, *pectin methylesterase inhibitor* (*EgPMEI*), *EgPLL17/19/26*, *NOD26-like intrinsic protein* (*EgNIP*) *5;1/6;1* and *vacuolar invertase* (*EgVI*) *1*, displayed reduced expression in the treated plants.

Further hindering the cellular expansion process, numerous genes associated with the biosynthesis of cell wall components such as cellulose and hemicellulose were downregulated under water stress (Fig. 3B). In terms of cellulose biosynthesis, *alpha 1,4-glycosyltransferase* (*Eg1,4-GTF*), *cellulose synthase* (*EgCES*) *A4/A9*, *cellulose synthase-like* (*EgCSL*) *A11/D3*, *cobra-like* (*EgCOB, EgCOBL*) *7* and *fasciclin-like* (*EgFLA*) *9/12* were downregulated during drought, while only *EgCSLG3* and *irregular xylem* (*EgIRX*) *1* were upregulated. Likewise, the hemicellulose-related genes, *glycosyltransferase family* (*EgGT*) *61*, *EgIRX9/14* and *galactan synthase* (*EgGALS*) *1*, displayed decreased expression, as opposed to *UDP-xylose synthase* (*EgAUD*) *1* which was elevated in the treated plants.

### Transcription factor-mediated regulation

**Figure 4:**
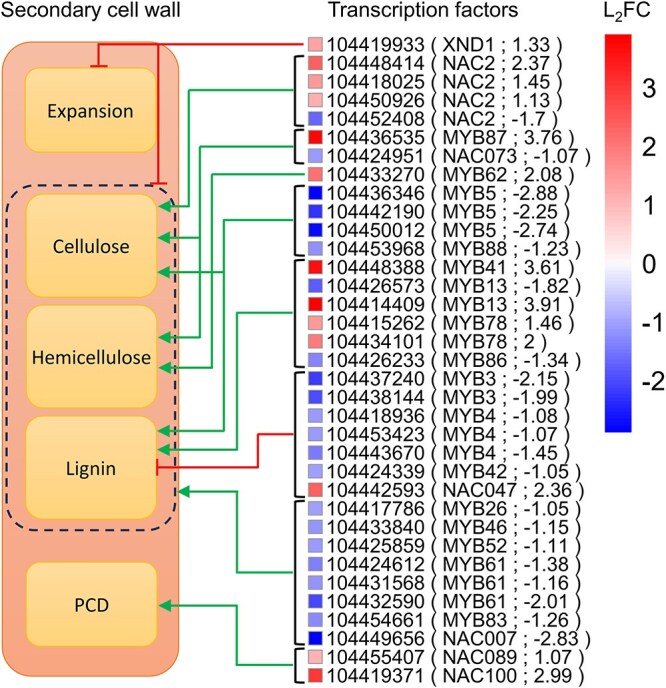
Significantly differentially expressed TFs with putative roles in the drought response in *Eucalyptus grandis*. The log_2_ fold change (L_2_FC) is with respect to the droughted condition. Red lines with blunt ends represent repression of the specific process, whereas green arrows depict the activation of the process. Transcription factors that positively or negatively regulate the cellulose, hemicellulose and lignin processes are those that point to the dotted black line. The heatmap cluster was generated in R v4.3.1 software (R Core Team 2022).

Two major families of TFs, principally the *NAC* (NAM, ATAF1,2 and CUC2) and *MYB* (Myeloblastosis) TFs implicated in governing fiber and vessel anatomy (Nakano et al. 2015), were detected (Fig. 4). In the droughted condition, *xylem NAC domain* (*EgXND*) *1*, associated with inhibiting cellular expansion and cell wall component biosynthesis, was upregulated. Water deprivation also increased the expression of *EgNAC089/100*, a regulator of programmed cell death. Many TFs promoting cellulose (*EgNAC2/073*, *EgMYB5/88*), hemicellulose (*EgNAC073*) and lignin (*EgMYB5/13/86/88*) biosynthesis were downregulated in the treated plants (Fig. 4). Similarly, the expression of *EgMYB26/46/52/61/83* and *NAC007*, which are known to support the synthesis of all the aforementioned cell wall components, was lower compared with the controls. Conversely, a few TFs promoting the synthesis of cellulose (*EgNAC2*, *EgMYB87*), hemicellulose (*EgMYB87/62*) and lignin (*EgMYB13/41/78*) were elevated during drought (Fig. 4). Meanwhile, transcripts of *EgMYB3/4/42* linked to the repression of lignin biosynthesis were less abundant in the treated plants, with only a single repressor TF (*EgNAC047*) showing elevated expression.

### Phenylpropanoid pathway flux prioritization

**Figure 5:**
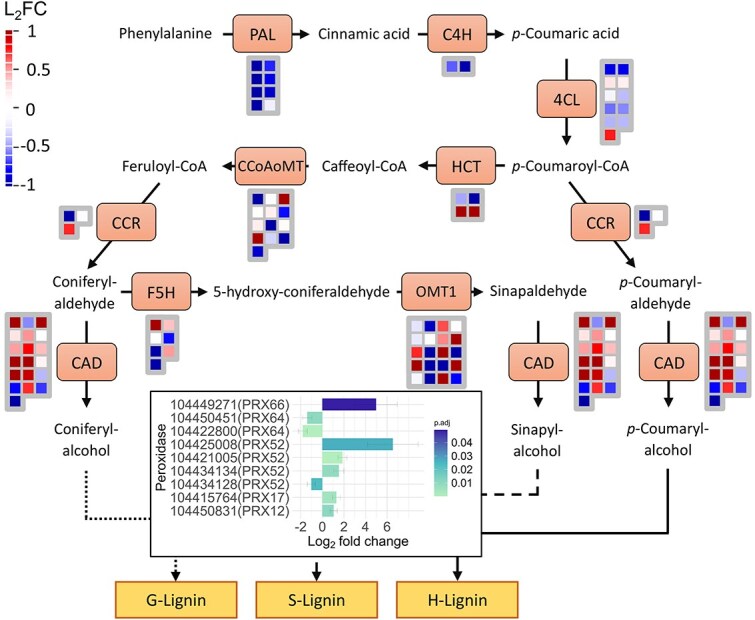
Phenylpropanoid pathway comparing the level of gene expression between control and droughted *Eucalyptus grandis*. The red gradient represents a positive fold change (upregulation), blue is a negative fold change (downregulation) and white is no difference. The log_2_ fold change (L_2_FC) of various peroxidases, responsible for the polymerization of G-, S- or H- lignin subunits is represented at the end of the pathway. Positive fold changes depict upregulation in the droughted condition and negative fold changes represent downregulation. Adjusted Benjamini–Hochberg corrected *P*-values are represented. The phenylpropanoid pathway was reconstructed using MapMan (Thimm et al. 2004) and R v4.3.1 software (R Core Team 2022), with consideration of the literature (Vanholme et al. 2019; Xiao et al. 2021) and the core lignin toolbox of *E. grandis* (Carocha et al. 2015).

Several genes involved in phenylpropanoid metabolism were differentially expressed between the control and droughted plants. *Phenylalanine ammonia-lyase* (*EgPAL*), *cinnamate 4-hydroxylase* (*EgC4H*) and *4-coumarate:CoA ligase* (*Eg4CL*) were downregulated in the treated plants, with *EgPAL* in particular showing a significant decrease (*P* < 0.01). However, further along the pathway, gene expression patterns changed, with the drought-stressed *E. grandis* showing a significant (*P* = 0.01) upregulation of genes encoding *cinnamyl alcohol dehydrogenase* (*EgCAD*; Fig. 5). As described in the literature, various peroxidases with roles in lignin polymerization were differentially expressed under our study conditions (Tokunaga et al. 2009; Fernández-Pérez et al. 2014; Cosio et al. 2017; M. Zhang et al. 2022). These were predominantly upregulated during drought (*EgPRX12/17/52/66*), compared with control (*EgPRX52/64*) conditions (Fig. 5). Although no significant differences in the lignin composition or SG ratio were recorded between the conditions tested, it seems that the fractional lignin area (i.e. lignin per unit area) was significantly (*P* = 0.04) greater in the drought-adapted wood (Table 1).

## Discussion

Xylem is a complex tissue, crucial for the survival of many terrestrial plants. This structure is also remarkably plastic, governed by intricate physiological and molecular processes that respond to several environmental factors. Our findings have demonstrated that a cascade of events is set in motion during xylogenesis that most significantly impacts cell size to promote hydraulically safer wood in drought-stressed *E. grandis*.

### Short- and long-term adaptations to drought in E. grandis

In the current study, *E. grandis* displayed an ability to regulate *g_sw_* in response to the water deficit imposed (Fig. 1A). This acute stomatal control, often observed in isohydric plants (Saunders and Drew 2022), means that at reduced *g_sw_*, slower growth could occur, with a greater margin of hydraulic safety in our droughted treatment (Fig. 1B, Table 1). Preserving gaseous exchange for photosynthesis as long as possible is likely a crucial element in the short-term adaptive response in our treated plants since the newly forming, drought-adapted wood requires photosynthates for formation to occur. Although stomatal closure can reduce the possibility of cavitation by regulating the water potential within the conduits, this response is not sustainable in the long term and thus emphasizes the importance of xylem anatomical adaptations.

Reduced *g_sw_* may cripple CO_2_ assimilation and possibly bottleneck photosynthate production and since these resources become scarcer over prolonged water deficit, the treated plants will require adaptations in the wood anatomy to accommodate for this. Indeed, adaptations of the xylem fibers and vessels occurred in response to the drought imposed, with the plants producing smaller, more wall-biased cells of a higher density (Table 1). The implications of a reduced cellular lumen in vessels are associated with improved hydraulic safety in *Eucalyptus* (Saunders and Drew 2022), as these are typically more implosion resistant and possess reduced pitted wall area, thereby reducing the probability of embolisms spreading (Zwieniecki and Secchi 2015; Jacobsen et al. 2019). Our study validates at least one of these mechanisms, whereby the implosion resistance was shown to be improved in smaller vessels produced during drought (Table 1). Despite being beneficial during water deficit, we found that these hydraulic safety features came at the expense of theoretical hydraulic conductance, a scenario that has been highlighted in the literature (Venturas et al. 2017; Rodriguez-Zaccaro and Groover 2019). To partially compensate for this loss of hydraulic conductance, the droughted plants in our study produced a greater number of vessels, as expected under drought which creates a functionally redundant safety mechanism, such that a higher number of vessels can conduct water at any given time (Islam et al. 2018; Mrad et al. 2021). The effect of fiber size and density on hydraulic safety has been explored to a considerably lower degree. However, there have been suggestions that smaller fibers at a higher density, as observed in our drought treated plants, can provide reinforcement to vessels to prevent microfracture-induced air seeding (Dória et al. 2018; Lourenço et al. 2022).

Given the context of the drought, *E. grandis* plants in our study did not enter a state of complete dormancy but rather adapted their physiology to enable growth at a reduced level. Understanding the changes in wood anatomy in these trees provides clues to the underpinnings of drought adaptation. For instance, the reduced hydraulic conductance of smaller vessels is likely acting in concert with stomatal regulation to fine-tune water relations within the entire plant, allowing stomata to stay open under drought and avoiding carbon starvation. Evidently, our findings suggest that the *E. grandis* short-term response to drought includes a reduction in the *g_sw_* value to reduce immediate water loss and tension in the xylem. Subsequently, smaller implosion and embolism-resistant cells are formed to enable physiological processes to continue in our treated plants, albeit at severely reduced rates (Badel et al. 2015).

Given that isohydric *Eucalyptus* species can regulate transpiration to stabilize the water potential in the xylem, they often require a lower degree of xylem plasticity or safety. This is because the tight control of stomatal openings allows isohydric plants to finely regulate water loss, mitigating the need to produce hydraulically safer xylem in the short term. Unlike isohydric plants, anisohydric species lack tight stomatal control, necessitating the development of xylem that can withstand more negative water tensions (Z. Chen et al. 2021). While the plants in our study initially displayed isohydric stomatal control as an immediate response to drought, prolonged stress triggered a hydraulically safer xylem anatomy (Table 1). These findings suggest that *E. grandis* falls on a spectrum between typical isohydric and anisohydric behaviors. It is likely that the balance between isohydric stomatal control (Fig. 1A) and xylem plasticity optimizes growth during periods of water availability while mitigating the risk of runaway cavitation during drought.

### Impact of drought on the transcriptomic landscape of E. grandis

Given that the adaptations of wood anatomical properties are crucial for plant survival in response to climate, it is expected that these are under stringent spatiotemporal transcriptomic regulation (Ployet et al. 2019). Interpreting the transcriptome is necessary to gain an overview of the adaptive mechanisms employed by *E. grandis* in response to water shortage. GSEA offers a tool to identify significantly enriched gene ontologies that underpin these transcriptomic responses (Wu et al. 2021). During drought, we found that numerous stress ontologies related to ROS, heat, programmed cell death and hypersensitive responses were enriched (Fig. 2B). Prolonged drought has been shown to retard plant growth and photosynthesis and increase programmed cell death due to the buildup of ROS (Ahanger et al. 2017). Consistent with our findings, recent studies have demonstrated that molecular responses to ROS (i.e. scavenging or antioxidant molecule accumulation) are a necessary acclimation strategy to enhance plant endurance during water deficit (Ilyas et al. 2021). Downstream secondary metabolites, including terpenes, flavonoids and other phenolic compounds identified in the current analysis, have been shown to serve as antioxidant molecules to reduce oxidative damage (Nicolas-Espinosa et al. 2023). Furthermore, ontologies associated with the catabolic breakdown of numerous organic compounds were enriched in our treated plants, presumably providing the energy to sustain basic metabolic processes or for osmoregulation. Depletion of the cell cycle, cell wall remodeling and component biosynthesis ontologies during drought correspond with the reduction in stem growth compared with the control plants (Figs 1B and 2B; Zhang et al. 2021). Our results highlight the impact of water deprivation on the transcriptome of *E. grandis* which is possibly centered around the control of, or response to, ROS.

### Cellular expansion requires coordinated cell wall modification and component gene expression

Turgor-driven cell wall expansion is a complex developmental process that requires stringent coordination between the loosening of polymers and the incorporation of newly synthesized components to increase the cellular volume (Cosgrove 2018). Although this aspect of cellular development remains to be fully elucidated (Zhang et al. 2021), the present study sought to uncover some potential genetic role players governing xylem cell enlargement in *E. grandis* under drought conditions.

Upon exposure to drought, genes associated with the inhibition of cellular expansion such as *EgHYR1*, *EgUGT76F1*, *EgPAE* and *EgPME3* were found to be upregulated (Fig. 3A). In *A. thaliana* seedlings, *HYR1* and *UGT76F1* have been shown to function exclusively as expansion inhibitors through conjugation of hypostatin (activation) or auxin precursors (inactivation), respectively (Tóth and van der Hoorn 2010; Chen et al. 2020). Additionally, pectin deacetylation by *PAE* has been documented to lead to structural configurations that reduce digestibility and hence may have prevented cell wall loosening in our droughted plants (Gou et al. 2012). Contrary to these unidirectional mechanisms, pectin demethylesterification by *PME* can potentially either decrease or increase cell wall extensibility of the control or droughted plants, depending on whether a ‘blockwise’ or ‘random’ methylesterification pattern is produced (Qiu et al. 2021). Blockwise patterns occur when *PME* generates long stretches of free carboxyl groups on multiple consecutive pectin polymers that can form calcium bonds with one another to generate a rigid ‘egg box’ structure (Wormit and Usadel 2018). The stiffened pectin-calcium-pectin linkages may serve to strengthen cell adhesion properties in the middle lamella and could have hindered intrusive growth in our treated plants. Conversely, random demethylesterification of pectin promotes depolymerization by pectin lyase and may have facilitated cell wall loosening in our controls, leading to the formation of larger cells (Dauphin et al. 2022). Although still a topic of ongoing research, the regulation of methylesterification patterns is believed to be controlled through the action of *PMEIs* (Wormit and Usadel 2018). A study by Kunieda et al. (2013) found that the inhibition of an unidentified *PME* by *PMEI6* produced a demethylesterification pattern suitable for *PRX36* anchoring, which in turn promoted localized loosening. Interestingly, the downregulation of *EgPMEIs* and *EgPRX36* was found to occur exclusively in our droughted plants. This suggests that the interaction between pectin methylesterase and its inhibitor is necessary for cell enlargement (Fig. 3A and B). Likewise, reduced expression of *EgGALS1*, indirectly responsible for the synthesis of pectic galactans, was observed in our treated *E. grandis* and may have hindered cellular growth (Ebert et al. 2018). However, whether the *PME-PMEI* interaction controls enlargement through random pattern-induced depolymerization or peroxidase-mediated loosening remains to be determined.

In our study, the expression of numerous genes that encourage cell expansion via modifications to cellulose in the cell wall matrix was identified in the controls (Fig. 3A and B). For instance, cellulose degradation by *EgCEL5* or remodeling of cellulose-hemicellulose (i.e. xyloglucans and xylans) crosslinks by *EgXYLs*, *EgTBLs* and *EgXTHs* may have manifested in the formation of larger cells in our control xylem (Alvarez et al. 2006; Chukhchin et al. 2020; Qaseem and Wu 2020; Li et al. 2021; Kabir et al. 2023). Furthermore, cellulose slippage can be induced by expansins, which disrupt the covalent bonds between adjacent cellulose microfibrils (Samalova et al. 2022). Although most were downregulated, we observed that *EXLB1/B2* displayed elevated expression in the treated plants, reflecting the need for xylogenic fibers and vessels in the differentiation zone to expand, even during drought. Furthermore, the enlargement of xylem cells in the treated plants appears to be associated with the action of callose degradation by *EgBG1* (Perrot et al. 2022). In our controls, cellular rearrangements involving *EgMAP65s* (Mao et al. 2006; Lucas et al. 2011), the conversion of sucrose into hexose, glucose and fructose by *EgVI1* and a constant supply of boron by *EgNIP5;1* or *EgNIP6;1* (Funakawa and Miwa 2015; Chi et al. 2020; Tanaka and Fujiwara 2021) may indirectly contribute to cellular expansion.

The growing cell wall matrix requires freshly synthesized components such as cellulose and hemicellulose (Cosgrove 2018). Consistent with the smaller cells in the xylem of treated plants, many genes responsible for cellulose synthesis were downregulated, including *Eg1,4-GTF* (Daras et al. 2021), *EgCESAs* (Polko and Kieber 2019), *EgCSLs* (Liu et al. 2021), *EgCOBLs* (Lampugnani et al. 2019) and *EgFLAs* (MacMillan et al. 2015). Interestingly, several *COBRA*, *COBRA*-like and *Fasciclin*-like genes have been implicated in the control of directional cell expansion via orientating the cellulose microfibril placement into the developing cell wall (Lin et al. 2022; Sajjad et al. 2023). Furthermore, we found that only *EgCSLG3* and *EgIRX1* were upregulated during drought (Fig. 3B), which suggests a role for these cellulose synthases in the drought-adaptive response, possibly altering the cellulose composition or structure (Liu et al. 2020; L. Zhang et al. 2022). In our water stressed plants, it seems that the vast majority of xylan biosynthetic genes such as *EgIRX9/14* and *EgGT61* were downregulated, with the exception of *EgAUD1*, suggesting an attenuated level of cell wall remodeling (Zhong et al. 2022; Anders et al. 2023). Our findings strongly suggest that cell enlargement is primarily controlled through the metabolism of cellulose and pectin present in the cell wall matrix and that these are potentially crucial elements that underpin the wood anatomical adaptive response in *E. grandis*.

### Hierarchical control of cellular growth by TFs

An integral part of xylem cell regulation in trees is the expression of TFs belonging to the NAC and MYB families (Nakano et al. 2015). In comparison with the control plants under study, relatively few of the TFs that promote the synthesis of cellulose (*EgNAC2*, *EgMYB87*) and xylan (*EgMYB87/62*) were upregulated during drought (Fig. 4; Fujiwara et al. 2014; Srivastava et al. 2023). Rather, we found that processes such as lignification (*EgMYB13/41/78*) and programmed cell death (*EgNAC089/100*) seem to be prioritized (Kosma et al. 2014; Yang et al. 2014  2023; Serk et al. 2015; Ma et al. 2021). This is reiterated by the fact that numerous isoforms of *EgMYB3/4/42*, known to inhibit lignification, were downregulated in the treated plants (Ma and Constabel 2019; Xiao et al. 2021). Lignification and programmed cell death are terminal stages during the development of tracheary elements and our findings may suggest that a more rapid differentiation process was occurring during drought. Considering that we observed a reduced cell size and attenuation of remodeling-related genes during water deficit (Fig. 3A and B, Table 1), this expedited differentiation could stem from a shorter cellular enlargement period. In support of this mechanism is the upregulation of *EgXND1* in our droughted plants, which has been shown to simultaneously inhibit cell expansion and the synthesis of cell wall components (Zhong et al. 2021). The stimulation of component biosynthesis by TFs including *EgMYB5/13/26/46/52/61/83/88* (Serk et al. 2015; Geng et al. 2018; Xie et al. 2018; Wei et al. 2019; K. Chen et al. 2021, Xiao et al. 2021) and *EgNAC2/073/007* (Zhuo et al. 2018; Qi et al. 2022; Srivastava et al. 2023) in our controls corroborates our wood anatomical and functional analysis, since the larger cells (Table 1) were found to be associated with a higher activity of cellulose and hemicellulose remodeling genes (Fig. 3A and B).

### Prioritization of lignin during drought

The phenylpropanoid pathway is a key supplier of precursors required for lignin biosynthesis that forms a major component of the SCW (Vanholme et al. 2019). Many of the genes that belong to the ‘core lignin toolbox’ in *E. grandis* have been detected in the present study (Carocha et al. 2015). Although the early stages of the phenylpropanoid pathway did not show any significant perturbation under drought, the later stages responsible for the biosynthesis of various monolignols were upregulated (Fig. 5). Particularly, a significant increase in *EgCAD* expression could have prioritized the conversion of coniferylaldehyde, sinapaldehyde and *p*-coumarylaldehyde into G-, S- and H- lignin subunits, respectively. These findings indicate that metabolites in the phenylpropanoid pathway may be directed toward producing structural lignin for incorporation into the SCW. Interestingly, the expression of *EgPRX12/17/52/66* that catalyzes oxidative polymerization of lignin monomers was upregulated in our water stressed plants. Studies using *A. thaliana* mutant lines and *GUS* reporter gene fusions have demonstrated the roles of *PRX17*, *PRX52* and *PRX66* in the lignification of vascular tissues (Tokunaga et al. 2009; Fernández-Pérez et al. 2014; Cosio et al. 2017). Additionally, the overexpression of *EjPRX12* in *A. thaliana* has been shown to enhance lignin accumulation in the stem (Zhang, Shi, et al. 2022). Although these processes did not result in an elevated lignin composition in the wood per se, we propose that the phenylpropanoid derivatives are likely being prioritized for lignin production to retain control-like levels. This is crucial as lignin is an essential component in embolism and implosion resistance, by providing strength and rigidity to the SCW. Another aspect to consider is that the significantly elevated density and fractional cell wall area (i.e. per cross-sectional area) in the wood of our drought treated plants resulted in an increased lignin content on a per unit area basis and this may have enhanced the strength and water relations properties of the stem (Han et al. 2022). Despite our findings, lignin composition is often a feature that can increase in response to drought and perhaps given an extended growth period, these differences in lignin prioritization in our study may have eventually translated into compositional differences (Han et al. 2022).

## Conclusion

The controlled nature of our experiments in combination with high-resolution wood anatomy and transcriptomics data has provided a unique perspective of the short- and long-term responses of *E. grandis* to drought. Initially, the eucalypts displayed an isohydric stomatal response to mitigate water loss and maintain carbon assimilation during water deficit. This facilitated the formation of smaller high-density cells in the xylem that offered enhanced implosion resistance and functional redundancy. These anatomical changes were shown to correspond with the downregulation of genes responsible for polysaccharide biosynthesis and cellulose-hemicellulose loosening within the cell wall matrix, supposedly triggered by ROS. Similarly, MYB and NAC TFs suggest that the cell differentiation processes are expedited via a shorter elongation period, which may also function to conserve resources when water is limited. Upon drought exposure, the upregulation of *EgCAD* in the phenylpropanoid pathway suggests a flux that favors lignin production. This was reflected in an increased lignin content per unit area in the higher-density wood. These findings have provided a comprehensive overview of how cell enlargement is regulated in *E. grandis*, to produce xylem features that improve hydraulic safety. This comparative transcriptomics approach has established a clear relationship between the molecular network components and the drought anatomical phenotype adopted by *E. grandis* in response to water stress, which will serve as an essential foundation for increasingly realistic models of wood development, in relation to climate. Interesting gene sets and their association with hydraulic safety features can serve as a viable marker selection criterion for ideal tree provenances, suited to a particular environment to maximize yields through improved survivability.

## Supplementary Material

Table_S1_diameter_tpae068

Table_S2_height_tpae068

Table_S3_gsw_tpae068

Table_S4_lignin_tpae068

Table_S5_qwa_c_tpae068

Table_S6_qwa_d_tpae068

Table_S7_ir_tpae068

Table_S8_gsea_tpae068

Table_S9_degs_tpae068

Code_S1_physiology_tpae068

Code_S2_qwa_tpae068

Code_transcriptomics_tpae068

Method_S1_pilot_tpae068

Method_S2_ctab_tpae068

Method_S3_qc_tpae068

## Data Availability

The R code associated with the current article is available as supplementary materials and in the GitHub repository https://github.com/Rafael-Keret/Eucalyptus_transcriptomic_analysis. The *E. grandis* microsection scans used to generate quantitative wood anatomy data are available on Zenodo: https://doi.org/10.5281/zenodo.8246235, https://doi.org/10.5281/zenodo.8245566. The raw sequence reads are available at the National Centre for Biotechnology Information (NCBI); PRJNA1012834.
