# Correction to: Xylem cell size regulation is a key adaptive response to water deficit in *Eucalyptus grandis*

**DOI:** 10.1093/treephys/tpae108

**Published:** 2024-08-20

This is a correction to: Rafael Keret, David M Drew, Paul N Hills, Xylem cell size regulation is a key adaptive response to water deficit in Eucalyptus grandis, *Tree Physiology*, Volume 44, Issue 7, July 2024, tpae068, https://doi.org/10.1093/treephys/tpae068

The originally published version of the manuscript contained errors in the presentation of units cell mm^−2^ and cells mm^−2^.

The error occurred in Table 1, in the column entitled “Property”, where the units for “Density” were given as (cells/mm^2^) and (cells mm^2^).

Additionally, the error was present in the following sentence in the Results section:

Under drought conditions, a significant (*P* < 0.01) increase in cellular density from 5443.0 to 6239.8 cell mm^2^ for fibers and 63.89 to 88.24 cell mm^2^ in vessels (*P* = 0.01) increased the fractional cell wall area by 2.63%.

The Publisher apologizes for these errors, which have been corrected.

