# Supplementary material for: Xylem cell size regulation is a key adaptive response to water deficit in Eucalyptus grandis
Source: Tree Physiol. 2024 Jun 18;44(7):tpae068. doi: 10.1093/treephys/tpae068 (PMC11247191; doi:10.1093/treephys/tpae068)
Supplement: Code_S1_physiology_tpae068 [file code_s1_physiology_tpae068.pdf]

Code S1: Physiological responses of *Eucalyptus grandis* in response to water deficit

Rafael Keret

2024-02-13

STOMATAL CONDUCTANCE ANALYSIS

(1) Load packages

```
library("dplyr")
library("ggplot2")
library("tidyr")
library("emmeans")
library("car")
```

(2) Import Licor 600 dataframe and check normality

```
Licor_data <- read.table("./Data/Licor600/Table_S3_gsw.csv", sep = ",", skip = 1,
                         header = T)
```

(3) Normality (Shapiro-Wilks)

```
Licor_data %>%
  group_by(Treatment, Day) %>%
  summarise(p.value = shapiro.test(log(gsw))$p.value) %>%
  print(n = 32)
```

(4) Calculate the mean stomatal conductance and SE per day per treatment

```
SC_mean <- Licor_data %>%
  group_by(Day, Treatment) %>%
  summarise(gsw_mean = mean(gsw),
            gsw_SE = sd(gsw)/sqrt(length(Treatment)))
```

(5) Perform a repeated measures ANOVA (time series data) and Tukeys post hoc
Set the Days as a factor since there was correlation between the predictor and intercept in the model.

```
Licor_data$Day <- factor(Licor_data$Day)
contrasts(Licor_data$Day) <- contr.sum(length(levels(Licor_data$Day)))
```

(6) Test the effect of treatment and days on stomatal conductance (gsw)
The dependent variable (gsw) was square root (sqrt) transformed.

```
anova <- aov(sqrt(gsw) ~ Treatment * Day, data = Licor_data)
summary(anova)
```

(7) Test model assumptions

Normality

```
hist(residuals(anova))
plot(anova, which = 2)
```

Homogeneity of the variance: Residuals versus fitted values

```
plot(anova, which = 1)
leveneTest(residuals(anova) ~ Treatment, data = Licor_data)
```

(8) Tukeys HSD post hoc test, comparisons between treatments on the same day

```
Tukeys <- emmeans(anova, ~ Treatment | Day, contr = "tukey")

print(Tukeys)
```

(9) Extract the daily pairwise comparisons and adjusted p-values

```
Tukeys_post_hoc <- summary(contrast(Tukeys, interaction = "pairwise", adjust = "tukey"))
```

(10) Create a line plot with error bars

```
SC_plot <- ggplot(SC_mean, aes(x = Day, y = gsw_mean)) +
  geom_line(aes(linetype = Treatment, group = Treatment)) + geom_point(aes()) +
  geom_errorbar(aes(x = Day, ymin = gsw_mean - gsw_SE, ymax = gsw_mean + gsw_SE),
              width = 0.4, colour = "black", alpha = 0.9) +
  xlab("Day") + ylab(expression(g[sw] ~ (mmol. ~ m^-2 ~ .s^-1))) +
  theme(panel.background = element_rect(fill = "white"),
        panel.grid.major = element_blank(),
        panel.grid.minor = element_blank(),
        axis.line = element_line(color = "black")) +
  theme(axis.title = element_text(size = 20),
        axis.text = element_text(size = 20)) +
  theme(legend.title = element_text(size = 18),
        legend.text = element_text(size = 18)) +
  geom_text(data = SC_mean %>% filter(Treatment == "C") %>%
              mutate(label = ifelse(Day == 0, "**", "***")),
            aes(label = label),
            vjust = -1.3, hjust = 0.45, color = "black", size = 5)
```

STEM DIAMETER

(1) Load packages

```
library("dplyr")
library("ggplot2")
library("tidyr")
library("emmeans")
library("nortest")
```

(2) Import stem diameter dataframe and calculate relative SD (or plant growth)

```
Stem_diameter <- read.table("./Data/Physiology/Table_S1_diameter.csv", sep = ",", skip = 1,
                            header = T)
```

(3) Calculate change in stem diameter over time (i.e. growth)

```
Relative_SD <- Stem_diameter %>%
  group_by(Treatment) %>%
  arrange(Day) %>%
  mutate(Relative_SD_mm = Stem_diameter_mm - Stem_diameter_mm [Day == "0"])
```

(4) Check normality (Shapiro-Wilks)
Skip measurements at Day 0, because these will be zero and hence have no distribution.
Will get a warning message due to sqrt of some zero values on day 3.

```
Relative_SD %>%
  group_by(Treatment, Day) %>%
  filter(Day != 0) %>%
  summarise(p.value = shapiro.test(sqrt(Relative_SD_mm))$p.value) %>%
  print(n = 32)
```

(5) Calculate the mean relative SD and SE per day per treatment

```
Relative_SD_mean <- Relative_SD %>%
  group_by(Day, Treatment) %>%
  summarise(Relative_SD_mean = mean(Relative_SD_mm),
            Relative_SD_SE = sd(Relative_SD_mm)/sqrt(length(Treatment)))
```

(6) Perform a repeated measures ANOVA (time series data) and Tukeys post hoc
Set the Days as a factor.

```
Relative_SD$Day <- factor(Relative_SD$Day)
contrasts(Relative_SD$Day) <- contr.sum(length(levels(Relative_SD$Day)))
```

(7) Test the effect of treatment and day on stem diameter (SD)
The dependent variable (SD) was square root (sqrt) transformed.
Dropping first day from the model as all values will be 0, and thus statistics is not applicable.

```
anova <- aov(sqrt(Relative_SD_mm) ~ Treatment * Day, data = Relative_SD[Relative_SD$Day != 0, ])
summary(anova)
```

(8) Test model assumptions

Normality

```
hist(residuals(anova))
plot(anova, which = 2)
```

Homogeneity of the variance: Residuals versus fitted values

```
plot(anova, which = 1)
```

(9) Tukeys HSD post hoc test, comparisons between treatments on the same day

```
Tukeys <- emmeans(anova, ~ Treatment | Day, contr = "tukey")

print(Tukeys)
```

(10) Extract the daily pairwise comparisons and adjusted p-values

```
Tukeys_post_hoc <- summary(contrast(Tukeys, interaction = "pairwise", adjust = "tukey"))
```

(11) Create a line plot with error bars

```
SD_plot <- ggplot(Relative_SD_mean, aes(x = Day, y = Relative_SD_mean)) +
  geom_line(aes(linetype = Treatment, group = Treatment)) + geom_point(aes()) +
  geom_errorbar(aes(x = Day, ymin = Relative_SD_mean - Relative_SD_SE,
                    ymax = Relative_SD_mean + Relative_SD_SE),
                width = 0.4, colour = "black", alpha = 0.9) +
  xlab("Day") + ylab(expression(Relative ~ SD ~ (mm))) +
  theme(panel.background = element_rect(fill = "white"),
        panel.grid.major = element_blank(),
        panel.grid.minor = element_blank(),
        axis.line = element_line(color = "black")) +
  theme(axis.title = element_text(size = 20),
        axis.text = element_text(size = 20)) +
  theme(legend.title = element_text(size = 18),
        legend.text = element_text(size = 18)) +
  geom_text(data = Relative_SD_mean %>%
              filter(Treatment == "C") %>%
              mutate(label = case_when(
                Day == 0 ~ "NS",
                Day == 3 ~ "NS",
                Day == 6 ~ "NS",
                Day == 9 ~ "NS",
                Day == 12 ~ "*",
                Day == 15 ~ "**",
                TRUE ~ "***"
              )),
            aes(label = label),
            vjust = -1.3, hjust = 0.45, color = "black", size = 5)
```

PLANT HEIGHT

(1) Load packages

```
library("dplyr")
library("ggplot2")
library("ggpubr")
```

(2) Import plant height dataframe

```
Plant_height <- read.table("./Data/Physiology/Table_S2_height.csv", sep = ",", skip = 1,
                           header = T)
```

(3) Calculate the overall mean change in PH (i.e. growth) and SE

```
PH_mean <- Plant_height %>%
  group_by(Treatment) %>%
  summarise(PH_mean = mean(Change_height_cm),
            PH_SE = sd(Change_height_cm)/sqrt(length(Treatment)))
```

(4) Checking normality

```
shapiro.test(Plant_height$Change_height_cm [Plant_height$Treatment == "C"])
shapiro.test(Plant_height$Change_height_cm [Plant_height$Treatment == "D"])
```

(5) Significance / hypothesis testing

```
wilcox.test(Plant_height$Change_height_cm [Plant_height$Treatment == "C"],
            Plant_height$Change_height_cm [Plant_height$Treatment == "D"])
```

(6) Construct graph

```
PH_graph <- ggbarplot(PH_mean, x = "Treatment", y = "PH_mean", fill = "Treatment") +
  ggtitle("Growth over 30-d") +
  theme(plot.title = element_text(hjust = 0.5, vjust = 2.5)) +
  ylab("Change in height (cm)") + theme(strip.background = element_blank(),
                                        strip.placement = "outside") +
  theme(legend.position = "none") +
  geom_errorbar(aes(x = Treatment, ymin = PH_mean - PH_SE, ymax = PH_mean + PH_SE),
                width = 0.4, colour = "black", alpha = 0.9, size = 0.02) +
  scale_fill_manual(values = c("grey90", "grey50")) +
  theme(axis.text.x = element_text(colour = "black", size = 20, angle = 0,
                                   margin = margin(t = 3))) +
  theme(axis.text.y = element_text(colour = "black", size = 20, angle = 0,
                                   margin = margin(r = 3))) +
  theme(axis.title.x = element_blank()) +
  theme(axis.title.y = element_text(size = 20, margin = margin(r = 3))) +
  theme(text = element_text(family = "Aerial", size = 20)) +
  geom_signif(comparisons = list(c("C", "D")), map_signif_level = TRUE,
              annotations = c("***"), y = 16)
```

LIGNIN CONTENT

(1) Load packages

```
library("dplyr")
library("ggplot2")
library("ggpubr")
```

(2) Import lignin dataframe

```
Lignin <- read.table("./Data/Physiology/Table_S4_lignin.csv", sep = ",", skip = 1,
                     header = T)
```

(3) Calculate the overall mean lignin and SG ratio as well as their respective SE

```
Lignin_mean <- Lignin %>%
  group_by(Treatment) %>%
  summarise(Soluble_lignin = mean(Soluble_lignin_.),
            Klason_lignin = mean(Klason_lignin_.),
            Total_lignin = mean(Total_lignin_.),
            SG_lignin = mean(SG_ratio),
            Soluble_lignin_SE = sd(Soluble_lignin_.)/sqrt(length(Treatment)),
            Klason_lignin_SE = sd(Klason_lignin_.)/sqrt(length(Treatment)),
            Total_lignin_SE = sd(Total_lignin_.)/sqrt(length(Treatment)),
            SG_lignin_SE = sd(SG_ratio)/sqrt(length(Treatment)))
```

(4) Checking normality

Soluble lignin percentage

```
shapiro.test(Lignin$Soluble_lignin_. [Lignin$Treatment == "C"])
shapiro.test(Lignin$Soluble_lignin_. [Lignin$Treatment == "D"])
```

Klasons lignin percentage

```
shapiro.test(Lignin$Klason_lignin_. [Lignin$Treatment == "C"])
shapiro.test(Lignin$Klason_lignin_. [Lignin$Treatment == "D"])
```

Total lignin percentage

```
shapiro.test(Lignin$Total_lignin_. [Lignin$Treatment == "C"])
shapiro.test(Lignin$Total_lignin_. [Lignin$Treatment == "D"])
```

SG ratio

```
shapiro.test(Lignin$SG_ratio [Lignin$Treatment == "C"])
shapiro.test(Lignin$SG_ratio [Lignin$Treatment == "D"])
```

(5) Significance / hypothesis testing

Soluble lignin percentage

```r
t.test(Lignin$Soluble_lignin_. [Lignin$Treatment == "C"],
       Lignin$Soluble_lignin_. [Lignin$Treatment == "D"])
```

Klasons lignin percentage

```r
t.test(Lignin$Klason_lignin_. [Lignin$Treatment == "C"],
       Lignin$Klason_lignin_. [Lignin$Treatment == "D"])
```

Total lignin percentage

```r
t.test(Lignin$Total_lignin_. [Lignin$Treatment == "C"],
       Lignin$Total_lignin_. [Lignin$Treatment == "D"])
```

SG ratio

```r
t.test(Lignin$SG_ratio [Lignin$Treatment == "C"],
       Lignin$SG_ratio [Lignin$Treatment == "D"])
```

(6) Construct graphs

Total lignin

```r
L_graph <- ggbarplot(Lignin_mean, x = "Treatment", y = "Total_lignin", fill = "Treatment") +
  ggtitle("Lignin content") +
  theme(plot.title = element_text(hjust = 0.5, vjust = 2.5)) +
  ylab("Klason lignin %") + theme(strip.background = element_blank(),
                                  strip.placement = "outside") +
  theme(legend.position = "none") +
  geom_errorbar(aes(x = Treatment, ymin = Total_lignin - Total_lignin_SE,
                    ymax = Total_lignin + Total_lignin_SE),
                width = 0.4, colour = "black", alpha = 0.9, size = 0.02) +
  scale_fill_manual(values = c("grey90", "grey50")) +
  theme(axis.text.x = element_text(colour = "black", size = 13, angle = 0,
                                   margin = margin(t = 3))) +
  theme(axis.text.y = element_text(colour = "black", size = 13, angle = 0,
                                   margin = margin(r = 3))) +
  theme(axis.title.x = element_blank()) +
  theme(axis.title.y = element_text(size = 15, margin = margin(r = 3))) +
  theme(text = element_text(family = "Aerial", size = 15)) +
  geom_signif(comparisons = list(c("C", "D")), map_signif_level = TRUE,
              annotations = c("NS"), y = 36)
```

SG ratio

```r
SG_graph <- ggbarplot(Lignin_mean, x = "Treatment", y = "SG_lignin", fill = "Treatment") +
  ggtitle("SG ratio") +
  theme(plot.title = element_text(hjust = 0.5, vjust = 2.5)) +
  ylab("S/G") + theme(strip.background = element_blank(), strip.placement = "outside") +
  theme(legend.position = "none") +
  geom_errorbar(aes(x = Treatment, ymin = SG_lignin - SG_lignin_SE,
```

```
                    ymax = SG_lignin + SG_lignin_SE),
                width = 0.4, colour = "black", alpha = 0.9, size = 0.02) +
  scale_fill_manual(values = c("grey90", "grey50")) +
  theme(axis.text.x = element_text(colour = "black", size = 13, angle = 0,
                                   margin = margin(t = 3))) +
  theme(axis.text.y = element_text(colour = "black", size = 13, angle = 0,
                                   margin = margin(r = 3))) +
  theme(axis.title.x = element_blank()) +
  theme(axis.title.y = element_text(size = 15, margin = margin(r = 3))) +
  theme(text = element_text(family = "Aerial", size = 15)) +
  geom_signif(comparisons = list(c("C", "D")), map_signif_level = TRUE,
              annotations = c("NS"), y = 2.95)
```

TEMPERATURE AND HUMIDITY

(1) Load packages

```
library("dplyr")
```

(2) Specify path to iButton data files and append into a single dataframe for the repeats

```
Path_to_csv <- dir("./Data/iButton",  pattern = ".csv", full.names = T)

iButton <- do.call(rbind, lapply(Path_to_csv, function(i) read.csv(i)))
```

(3) Calculate the mean temperature and humidity with SE

```
abiotic_conditions <- iButton %>%
  summarise(Temp_mean = mean(Temperature),
            Temp_SE = sd(Temperature),
            Humid_mean = mean(Humidity),
            Humid_SE = sd(Humidity))
```
