# Supplementary material for: Xylem cell size regulation is a key adaptive response to water deficit in Eucalyptus grandis
Source: Tree Physiol. 2024 Jun 18;44(7):tpae068. doi: 10.1093/treephys/tpae068 (PMC11247191; doi:10.1093/treephys/tpae068)
Supplement: Code_S2_qwa_tpae068 [file code_s2_qwa_tpae068.pdf]

Code S2: Comparing the xylem anatomy of *Eucalyptus grandis* subject to control and droughted watering regimes

Rafael Keret

2024-02-13

IMPORTING DATA

(1) Load tidyverse

```
library(tidyverse)
```

(2) Import QuPath raw csv data file and indicate tab ("/t") delimiter to separate the data into columns

```
Control_data <- read.table("./Data/input/Table_S5_qwa_c.csv", sep = ",", skip = 1,
                           header = T)
Droughted_data <- read.table("./Data/input/Table_S6_qwa_d.csv", sep = ",",skip = 1,
                             header = T)

Control_data$Class <- gsub(": Positive", "", Control_data$Class)
Droughted_data$Class <- gsub(": Positive", "", Droughted_data$Class)
```

(3) Selecting Fibers and Vessels from the dataframe

```
Control_data <- subset(Control_data, Class %in% c("Fibers", "Vessels"))
Droughted_data <- subset(Droughted_data, Class %in% c("Fibers", "Vessels"))
```

(4) Add cell wall area (CWA) and cell wall thickness (CWT) to the dataframe

```
Control_data$CWA <- Control_data$Cell..Area - Control_data$Nucleus..Area

Droughted_data$CWA <- Droughted_data$Cell..Area - Droughted_data$Nucleus..Area

Control_data$CWT <- ((Control_data$Cell..Max.caliper - Control_data$Nucleus..Max.caliper) +
                  (Control_data$Cell..Min.caliper - Control_data$Nucleus..Min.caliper)) / 2

Droughted_data$CWT <- ((Droughted_data$Cell..Max.caliper - Droughted_data$Nucleus..Max.caliper) +
                    (Droughted_data$Cell..Min.caliper - Droughted_data$Nucleus..Min.caliper)) / 2
```

(5) Write csv files for control and droughted data

```r
write.csv(Control_data, "./Data/output/Control_QWA_data.csv", row.names = FALSE)
write.csv(Droughted_data, "./Data/output/Droughted_QWA_data.csv", row.names = FALSE)
```

FIBER AND VESSEL PROPERTY ANALYSIS

(1) Calculate ROI size

Calculate ROI size (for cell density per unit area calculations)
Diameter = 480uM - 48uM = 432uM (removed region protruding into ROI)
Radius = 216uM
Area = 146074.377uM2 or 0.146074377mm2 or 1.46074377e-7m2

(2) Calculate the mean cell properties per slide / image (i.e. per tree sample)

```r
Control_mean <- Control_data %>%
  group_by(Image, Class) %>%
  summarise(CA_Mean = mean(Cell..Area),
            LA_Mean = mean(Nucleus..Area),
            LC_Mean = mean(Nucleus.Cell.area.ratio),
            CWA_Mean = mean(CWA),
            CWT_Mean = mean(CWT),
            Density = length(Class)/0.146074377)

Droughted_mean <- Droughted_data %>%
  group_by(Image, Class) %>%
  summarise(CA_Mean = mean(Cell..Area),
            LA_Mean = mean(Nucleus..Area),
            LC_Mean = mean(Nucleus.Cell.area.ratio),
            CWA_Mean = mean(CWA),
            CWT_Mean = mean(CWT),
            Density = length(Class)/0.146074377)
```

(3) Calculate the overall mean and SE for treatments (i.e. Control vs Droughted)

```r
Control_grouped <- Control_mean %>%
  group_by(Class) %>%
  summarise(CA_mean = mean(CA_Mean),
            LA_mean = mean(LA_Mean),
            LC_mean = mean(LC_Mean),
            CWA_mean = mean(CWA_Mean),
            CWT_mean = mean(CWT_Mean),
            Density_mean = mean(Density),
            CA_SE = sd(CA_Mean)/sqrt(36),
            LA_SE = sd(LA_Mean)/sqrt(36),
            LC_SE = sd(LC_Mean)/sqrt(36),
            CWA_SE = sd(CWA_Mean)/sqrt(36),
            CWT_SE = sd(CWT_Mean)/sqrt(36),
            Density_SE = sd(Density)/sqrt(36))

Control <- "C"
Control_grouped$Treatment <- Control
```

```r
Droughted_grouped <- Droughted_mean %>%
  group_by(Class) %>%
  summarise(CA_mean = mean(CA_Mean),
            LA_mean = mean(LA_Mean),
            LC_mean = mean(LC_Mean),
            CWA_mean = mean(CWA_Mean),
            CWT_mean = mean(CWT_Mean),
            Density_mean = mean(Density),
            CA_SE = sd(CA_Mean)/sqrt(36),
            LA_SE = sd(LA_Mean)/sqrt(36),
            LC_SE = sd(LC_Mean)/sqrt(36),
            CWA_SE = sd(CWA_Mean)/sqrt(36),
            CWT_SE = sd(CWT_Mean)/sqrt(36),
            Density_SE = sd(Density)/sqrt(36))

Droughted <- "D"
Droughted_grouped$Treatment <- Droughted

Property_summary <- bind_rows(Control_grouped, Droughted_grouped, id = NULL)
head(Property_summary)
```

CHECK NORMALITY

(1) Cell Area

Control

```r
shapiro.test(log(Control_mean$CA_Mean) [Control_mean$Class == "Fibers"])
shapiro.test(log(Control_mean$CA_Mean) [Control_mean$Class == "Vessels"])
```

Droughted

```r
shapiro.test(log(Droughted_mean$CA_Mean) [Droughted_mean$Class == "Fibers"])
shapiro.test(log(Droughted_mean$CA_Mean) [Droughted_mean$Class == "Vessels"])
```

(2) Lumen Area

Control

```r
shapiro.test(log(Control_mean$LA_Mean) [Control_mean$Class == "Fibers"])
shapiro.test(log(Control_mean$LA_Mean) [Control_mean$Class == "Vessels"])
```

Droughted

```r
shapiro.test(log(Droughted_mean$LA_Mean) [Droughted_mean$Class == "Fibers"])
shapiro.test(log(Droughted_mean$LA_Mean) [Droughted_mean$Class == "Vessels"])
```

(3) Lumen area to cell area Ratio

Control

```r
shapiro.test(Control_mean$LC_Mean [Control_mean$Class == "Fibers"])
shapiro.test(Control_mean$LC_Mean [Control_mean$Class == "Vessels"])
```

Droughted

```r
shapiro.test(Droughted_mean$LC_Mean [Droughted_mean$Class == "Fibers"])
shapiro.test(Droughted_mean$LC_Mean [Droughted_mean$Class == "Vessels"])
```

(4) Cell wall area

Control

```r
shapiro.test(Control_mean$CWA_Mean [Control_mean$Class == "Fibers"])
shapiro.test(Control_mean$CWA_Mean [Control_mean$Class == "Vessels"])
```

Droughted

```r
shapiro.test(Droughted_mean$CWA_Mean [Droughted_mean$Class == "Fibers"])
shapiro.test(Droughted_mean$CWA_Mean [Droughted_mean$Class == "Vessels"])
```

(5) Cell wall thickness

Control

```r
shapiro.test(Control_mean$CWT_Mean [Control_mean$Class == "Fibers"])
shapiro.test(Control_mean$CWT_Mean [Control_mean$Class == "Vessels"])
```

Droughted

```r
shapiro.test(Droughted_mean$CWT_Mean [Droughted_mean$Class == "Fibers"])
shapiro.test(Droughted_mean$CWT_Mean [Droughted_mean$Class == "Vessels"])
```

(6) Density per mm2

Control

```r
shapiro.test(log(Control_mean$Density) [Control_mean$Class == "Fibers"])
shapiro.test(Control_mean$Density [Control_mean$Class == "Vessels"])
```

Droughted

```r
shapiro.test(log(Droughted_mean$Density) [Droughted_mean$Class == "Fibers"])
shapiro.test(Droughted_mean$Density [Droughted_mean$Class == "Vessels"])
```

SIGNIFICANCE / HYPOTHESIS TESTING

(1) Cell Area

```r
t.test(log(Control_mean$CA_Mean) [Control_mean$Class == "Fibers"],
       log(Droughted_mean$CA_Mean) [Droughted_mean$Class == "Fibers"], paired = FALSE)
t.test(log(Control_mean$CA_Mean) [Control_mean$Class == "Vessels"],
       log(Droughted_mean$CA_Mean) [Droughted_mean$Class == "Vessels"], paired = FALSE)
```

(2) Lumen Area

```r
t.test(log(Control_mean$LA_Mean) [Control_mean$Class == "Fibers"],
       log(Droughted_mean$LA_Mean) [Droughted_mean$Class == "Fibers"], paired = FALSE)
t.test(log(Control_mean$LA_Mean) [Control_mean$Class == "Vessels"],
       log(Droughted_mean$LA_Mean) [Droughted_mean$Class == "Vessels"], paired = FALSE)
```

(3) Lumen area to cell area ratio

```r
t.test(Control_mean$LC_Mean [Control_mean$Class == "Fibers"],
       Droughted_mean$LC_Mean [Droughted_mean$Class == "Fibers"], paired = FALSE)
t.test(Control_mean$LC_Mean [Control_mean$Class == "Vessels"],
       Droughted_mean$LC_Mean [Droughted_mean$Class == "Vessels"], paired = FALSE)
```

(4) Cell wall area

```r
t.test(Control_mean$CWA_Mean [Control_mean$Class == "Fibers"],
       Droughted_mean$CWA_Mean [Droughted_mean$Class == "Fibers"], paired = FALSE)
t.test(Control_mean$CWA_Mean [Control_mean$Class == "Vessels"],
       Droughted_mean$CWA_Mean [Droughted_mean$Class == "Vessels"], paired = FALSE)
```

(5) Cell wall thickness

```r
t.test(Control_mean$CWT_Mean [Control_mean$Class == "Fibers"],
       Droughted_mean$CWT_Mean [Droughted_mean$Class == "Fibers"], paired = FALSE)
t.test(Control_mean$CWT_Mean [Control_mean$Class == "Vessels"],
       Droughted_mean$CWT_Mean [Droughted_mean$Class == "Vessels"], paired = FALSE)
```

(6) Density per mm2

```r
t.test(log(Control_mean$Density) [Control_mean$Class == "Fibers"],
       log(Droughted_mean$Density) [Droughted_mean$Class == "Fibers"], paired = FALSE)
t.test(Control_mean$Density [Control_mean$Class == "Vessels"],
       Droughted_mean$Density [Droughted_mean$Class == "Vessels"], paired = FALSE)
```

FIBER AND VESSEL PROPERTY GRAPHS

```r
library(ggpubr)
```

(1) Fiber graphs

```
Fiber_property <- subset(Property_summary, Class == "Fibers")
```

Cell area

```
CA_F <- ggbarplot(Fiber_property, x = "Treatment", y = "CA_mean", fill = "Treatment") +
  ggtitle("Fiber") +
  theme(plot.title = element_text(hjust = 0.5, vjust = 2.5)) +
  ylab("CA"~"("*mu*M^2*")") + theme(strip.background = element_blank(),
                                    strip.placement = "outside") +
  theme(legend.position = "none") +
  geom_errorbar(aes(x = Treatment, ymin = CA_mean - CA_SE, ymax = CA_mean + CA_SE),
                width = 0.4, colour = "black", alpha = 0.9, size = 0.02) +
  scale_fill_manual(values = c("grey90", "grey50")) +
  theme(axis.text.x = element_text(colour = "black", size = 13, angle = 0,
                                   margin = margin(t = 3))) +
  theme(axis.text.y = element_text(colour = "black", size = 13, angle = 0,
                                   margin = margin(r = 3))) +
  theme(axis.title.x = element_blank()) +
  theme(axis.title.y = element_text(size = 15, margin = margin(r = 3))) +
  theme(text = element_text(family = "Aerial", size = 15)) +
  geom_signif(comparisons = list(c("C", "D")), map_signif_level = TRUE,
              annotations = c("***"), y = 95)
```

Lumen area

```
LA_F <- ggbarplot(Fiber_property, x = "Treatment", y = "LA_mean", fill = "Treatment") +
  ggtitle("Fiber") +
  theme(plot.title = element_text(hjust = 0.5, vjust = 2.5)) +
  ylab("LA"~"("*mu*M^2*")") + theme(strip.background = element_blank(),
                                    strip.placement = "outside") +
  theme(legend.position = "none") +
  geom_errorbar(aes(x = Treatment, ymin = LA_mean - LA_SE, ymax = LA_mean + LA_SE),
                width = 0.4, colour = "black", alpha = 0.9, size = 0.02) +
  scale_fill_manual(values = c("grey90", "grey50")) +
  theme(axis.text.x = element_text(colour = "black", size = 13, angle = 0,
                                   margin = margin(t = 3))) +
  theme(axis.text.y = element_text(colour = "black", size = 13, angle = 0,
                                   margin = margin(r = 3))) +
  theme(axis.title.x = element_blank()) +
  theme(axis.title.y = element_text(size = 15, margin = margin(r = 3))) +
  theme(text = element_text(family = "Aerial", size = 15)) +
  geom_signif(comparisons = list(c("C", "D")), map_signif_level = TRUE,
              annotations = c("**"), y = 49)
```

Lumen area to cell area ratio

```
LC_F <- ggbarplot(Fiber_property, x = "Treatment", y = "LC_mean", fill = "Treatment") +
  ggtitle("Fiber") +
  theme(plot.title = element_text(hjust = 0.5, vjust = 2.5)) +
  ylab("LC ratio") + theme(strip.background = element_blank(),
                           strip.placement = "outside") +
  theme(legend.position = "none") +
```

```
  geom_errorbar(aes(x = Treatment, ymin = LC_mean - LC_SE, ymax = LC_mean + LC_SE),
                width = 0.4, colour = "black", alpha = 0.9, size = 0.02) +
  scale_fill_manual(values = c("grey90", "grey50")) +
  theme(axis.text.x = element_text(colour = "black", size = 13, angle = 0,
                                   margin = margin(t = 3))) +
  theme(axis.text.y = element_text(colour = "black", size = 13, angle = 0,
                                   margin = margin(r = 3))) +
  theme(axis.title.x = element_blank()) +
  theme(axis.title.y = element_text(size = 15, margin = margin(r = 3))) +
  theme(text = element_text(family = "Aerial", size = 15)) +
  geom_signif(comparisons = list(c("C", "D")), map_signif_level = TRUE,
              annotations = c("NS, p = 0.067"), y = 0.485)
```

Cell wall area

```
CWA_F <- ggbarplot(Fiber_property, x = "Treatment", y = "CWA_mean", fill = "Treatment") +
  ggtitle("Fiber") +
  theme(plot.title = element_text(hjust = 0.5, vjust = 2.5)) +
  ylab("CWA"~"("*mu*M^2*")") + theme(strip.background = element_blank(),
                                     strip.placement = "outside") +
  theme(legend.position = "none") +
  geom_errorbar(aes(x = Treatment, ymin = CWA_mean - CWA_SE, ymax = CWA_mean + CWA_SE),
                width = 0.4, colour = "black", alpha = 0.9, size = 0.02) +
  scale_fill_manual(values = c("grey90", "grey50")) +
  theme(axis.text.x = element_text(colour = "black", size = 13, angle = 0,
                                   margin = margin(t = 3))) +
  theme(axis.text.y = element_text(colour = "black", size = 13, angle = 0,
                                   margin = margin(r = 3))) +
  theme(axis.title.x = element_blank()) +
  theme(axis.title.y = element_text(size = 15, margin = margin(r = 3))) +
  theme(text = element_text(family = "Aerial", size = 15)) +
  geom_signif(comparisons = list(c("C", "D")), map_signif_level = TRUE,
              annotations = c("***"), y = 47)
```

Cell wall thickness

```
CWT_F <- ggbarplot(Fiber_property, x = "Treatment", y = "CWT_mean", fill = "Treatment") +
  ggtitle("Fiber") +
  theme(plot.title = element_text(hjust = 0.5, vjust = 2.5)) +
  ylab("CWT"~"("*mu*M*")") + theme(strip.background = element_blank(),
                                   strip.placement = "outside") +
  theme(legend.position = "none") +
  geom_errorbar(aes(x = Treatment, ymin = CWT_mean - CWT_SE, ymax = CWT_mean + CWT_SE),
                width = 0.4, colour = "black", alpha = 0.9, size = 0.02) +
  scale_fill_manual(values = c("grey90", "grey50")) +
  theme(axis.text.x = element_text(colour = "black", size = 13, angle = 0,
                                   margin = margin(t = 3))) +
  theme(axis.text.y = element_text(colour = "black", size = 13, angle = 0,
                                   margin = margin(r = 3))) +
  theme(axis.title.x = element_blank()) +
  theme(axis.title.y = element_text(size = 15, margin = margin(r = 3))) +
  theme(text = element_text(family = "Aerial", size = 15)) +
```

```
  geom_signif(comparisons = list(c("C", "D")), map_signif_level = TRUE,
              annotations = c("NS, p = 0.321"), y = 3.4)
```

Density

```
Density_F <- ggbarplot(Fiber_property, x = "Treatment", y = "Density_mean",
                       fill = "Treatment") +
  ggtitle("Fiber") +
  theme(plot.title = element_text(hjust = 0.5, vjust = 2.5)) +
  ylab(expression(Density ~ (cells/mm^2))) + theme(strip.background = element_blank(),
                                                   strip.placement = "outside") +
  theme(legend.position = "none") +
  geom_errorbar(aes(x = Treatment, ymin = Density_mean - Density_SE,
                    ymax = Density_mean + Density_SE),
                width = 0.4, colour = "black", alpha = 0.9, size = 0.02) +
  scale_fill_manual(values = c("grey90", "grey50")) +
  theme(axis.text.x = element_text(colour = "black", size = 13, angle = 0,
                                   margin = margin(t = 3))) +
  theme(axis.text.y = element_text(colour = "black", size = 13, angle = 0,
                                   margin = margin(r = 3))) +
  theme(axis.title.x = element_blank()) +
  theme(axis.title.y = element_text(size = 15, margin = margin(r = 3))) +
  theme(text = element_text(family = "Aerial", size = 15)) +
  geom_signif(comparisons = list(c("C", "D")), map_signif_level = TRUE,
              annotations = c("**"), y = 6500)
```

(2) Vessel graphs

```
Vessel_property <- subset(Property_summary, Class == "Vessels")
```

Cell area

```
CA_V <- ggbarplot(Vessel_property, x = "Treatment", y = "CA_mean", fill = "Treatment") +
  ggtitle("Vessel") +
  theme(plot.title = element_text(hjust = 0.5, vjust = 2.5)) +
  ylab("CA"~"("*mu*M^2*")") + theme(strip.background = element_blank(),
                                    strip.placement = "outside") +
  theme(legend.position = "none") +
  geom_errorbar(aes(x = Treatment, ymin = CA_mean - CA_SE, ymax = CA_mean + CA_SE),
                width = 0.4, colour = "black", alpha = 0.9, size = 0.02) +
  scale_fill_manual(values = c("grey90", "grey50")) +
  theme(axis.text.x = element_text(colour = "black", size = 13, angle = 0,
                                   margin = margin(t = 3))) +
  theme(axis.text.y = element_text(colour = "black", size = 13, angle = 0,
                                   margin = margin(r = 3))) +
  theme(axis.title.x = element_blank()) +
  theme(axis.title.y = element_text(size = 15, margin = margin(r = 3))) +
  theme(text = element_text(family = "Aerial", size = 15)) +
  geom_signif(comparisons = list(c("C", "D")), map_signif_level = TRUE,
              annotations = c("***"), y = 2960)
```

Lumen area

```
LA_V <- ggbarplot(Vessel_property, x = "Treatment", y = "LA_mean", fill = "Treatment") +
  ggtitle("Vessel") +
  theme(plot.title = element_text(hjust = 0.5, vjust = 2.5)) +
  ylab("LA"~"("*mu*M^2*")") + theme(strip.background = element_blank(),
                                    strip.placement = "outside") +
  theme(legend.position = "none") +
  geom_errorbar(aes(x = Treatment, ymin = LA_mean - LA_SE, ymax = LA_mean + LA_SE),
                width = 0.4, colour = "black", alpha = 0.9, size = 0.02) +
  scale_fill_manual(values = c("grey90", "grey50")) +
  theme(axis.text.x = element_text(colour = "black", size = 13, angle = 0,
                                   margin = margin(t = 3))) +
  theme(axis.text.y = element_text(colour = "black", size = 13, angle = 0,
                                   margin = margin(r = 3))) +
  theme(axis.title.x = element_blank()) +
  theme(axis.title.y = element_text(size = 15, margin = margin(r = 3))) +
  theme(text = element_text(family = "Aerial", size = 15)) +
  geom_signif(comparisons = list(c("C", "D")), map_signif_level = TRUE,
              annotations = c("***"), y = 2630)
```

Lumen area to cell area ratio

```
LC_V <- ggbarplot(Vessel_property, x = "Treatment", y = "LC_mean", fill = "Treatment") +
  ggtitle("Vessel") +
  theme(plot.title = element_text(hjust = 0.5, vjust = 2.5)) +
  ylab("LC ratio") + theme(strip.background = element_blank(),
                           strip.placement = "outside") +
  theme(legend.position = "none") +
  geom_errorbar(aes(x = Treatment, ymin = LC_mean - LC_SE, ymax = LC_mean + LC_SE),
                width = 0.4, colour = "black", alpha = 0.9, size = 0.02) +
  scale_fill_manual(values = c("grey90", "grey50")) +
  theme(axis.text.x = element_text(colour = "black", size = 13, angle = 0,
                                   margin = margin(t = 3))) +
  theme(axis.text.y = element_text(colour = "black", size = 13, angle = 0,
                                   margin = margin(r = 3))) +
  theme(axis.title.x = element_blank()) +
  theme(axis.title.y = element_text(size = 15, margin = margin(r = 3))) +
  theme(text = element_text(family = "Aerial", size = 15)) +
  geom_signif(comparisons = list(c("C", "D")), map_signif_level = TRUE,
              annotations = c("***"), y = 0.88)
```

Cell wall area

```
CWA_V <- ggbarplot(Vessel_property, x = "Treatment", y = "CWA_mean", fill = "Treatment") +
  ggtitle("Vessel") +
  theme(plot.title = element_text(hjust = 0.5, vjust = 2.5)) +
  ylab("CWA"~"("*mu*M^2*")") + theme(strip.background = element_blank(),
                                     strip.placement = "outside") +
  theme(legend.position = "none") +
  geom_errorbar(aes(x = Treatment, ymin = CWA_mean - CWA_SE, ymax = CWA_mean + CWA_SE),
                width = 0.4, colour = "black", alpha = 0.9, size = 0.02) +
  scale_fill_manual(values = c("grey90", "grey50")) +
  theme(axis.text.x = element_text(colour = "black", size = 13, angle = 0,
                                   margin = margin(t = 3))) +
```

```
  theme(axis.text.y = element_text(colour = "black", size = 13, angle = 0,
                                   margin = margin(r = 3))) +
  theme(axis.title.x = element_blank()) +
  theme(axis.title.y = element_text(size = 15, margin = margin(r = 3))) +
  theme(text = element_text(family = "Aerial", size = 15)) +
  geom_signif(comparisons = list(c("C", "D")), map_signif_level = TRUE,
              annotations = c("***"), y = 345)
```

Cell wall thickness

```
CWT_V <- ggbarplot(Vessel_property, x = "Treatment", y = "CWT_mean", fill = "Treatment") +
  ggtitle("Vessel") +
  theme(plot.title = element_text(hjust = 0.5, vjust = 2.5)) +
  ylab("CWT"~"("*mu*M*")") + theme(strip.background = element_blank(),
                                   strip.placement = "outside") +
  theme(legend.position = "none") +
  geom_errorbar(aes(x = Treatment, ymin = CWT_mean - CWT_SE, ymax = CWT_mean + CWT_SE),
                width = 0.4, colour = "black", alpha = 0.9, size = 0.02) +
  scale_fill_manual(values = c("grey90", "grey50")) +
  theme(axis.text.x = element_text(colour = "black", size = 13, angle = 0,
                                   margin = margin(t = 3))) +
  theme(axis.text.y = element_text(colour = "black", size = 13, angle = 0,
                                   margin = margin(r = 3))) +
  theme(axis.title.x = element_blank()) +
  theme(axis.title.y = element_text(size = 15, margin = margin(r = 3))) +
  theme(text = element_text(family = "Aerial", size = 15)) +
  geom_signif(comparisons = list(c("C", "D")), map_signif_level = TRUE,
              annotations = c("NS, p = 0.118"), y = 3.9)
```

Density

```
Density_V <- ggbarplot(Vessel_property, x = "Treatment", y = "Density_mean",
                       fill = "Treatment") +
  ggtitle("Vessel") +
  theme(plot.title = element_text(hjust = 0.5, vjust = 2.5)) +
  ylab(expression(Density ~ (cells/mm^2))) + theme(strip.background = element_blank(),
                                                   strip.placement = "outside") +
  theme(legend.position = "none") +
  geom_errorbar(aes(x = Treatment, ymin = Density_mean - Density_SE,
                    ymax = Density_mean + Density_SE),
                width = 0.4, colour = "black", alpha = 0.9, size = 0.02) +
  scale_fill_manual(values = c("grey90", "grey50")) +
  theme(axis.text.x = element_text(colour = "black", size = 13, angle = 0,
                                   margin = margin(t = 3))) +
  theme(axis.text.y = element_text(colour = "black", size = 13, angle = 0,
                                   margin = margin(r = 3))) +
  theme(axis.title.x = element_blank()) +
  theme(axis.title.y = element_text(size = 15, margin = margin(r = 3))) +
  theme(text = element_text(family = "Aerial", size = 15)) +
  geom_signif(comparisons = list(c("C", "D")), map_signif_level = TRUE,
              annotations = c("**"), y = 95)
```

(3) Creating grid plots

```
library(cowplot)

plot_grid(CA_F, CA_V, labels = c('A', 'B'), label_size = 12, label_y = 0.99, ncol = 2)

plot_grid(LA_F, LA_V, labels = c('A', 'B'), label_size = 12, label_y = 0.98, ncol = 2)

plot_grid(LC_F, LC_V, labels = c('A', 'B'), label_size = 12, label_y = 0.98, ncol = 2)

plot_grid(CWA_F, CWA_V, labels = c('A', 'B'), label_size = 12, label_y = 0.98, ncol = 2)

plot_grid(CWT_F, CWT_V, labels = c('A', 'B'), label_size = 12, label_y = 0.98, ncol = 2)

plot_grid(Density_F, Density_V, labels = c('A', 'B'), label_size = 12, label_y = 0.98,
          ncol = 2)
```

VESSEL IMPLOSION RESISTANCE

(1) Read in data

```
implosion <- read.table("./Data/input/Table_S7_vi.csv", sep = ",", skip = 1,
                        header = T)
```

(2) Implosion calculation

```
implosion$IR <- (implosion$Double_wall_thickness/implosion$Diameter_max)^2
```

(3) Calculate mean IR per treatment

```
IR_mean <- implosion %>%
  group_by(Treatment) %>%
  summarise(IR_mean = mean(IR),
            IR_SE = sd(IR)/sqrt(36))
```

(4) Checking normality
Shapiro-Wilks test

```
shapiro.test(implosion$IR [implosion$Treatment == "C"])
shapiro.test(implosion$IR [implosion$Treatment == "D"])
```

(5) Significance / hypothesis testing
Non-parametric

```
wilcox.test(implosion$IR [implosion$Treatment == "C"],
            implosion$IR [implosion$Treatment == "D"],
            alternative = "two.sided")
```

(6) Graphing
IR Plot

```
IR_graph <- ggbarplot(IR_mean, x = "Treatment", y = "IR_mean", fill = "Treatment") +
  ggtitle("Vessel") +
  theme(plot.title = element_text(hjust = 0.5, vjust = 2.5)) +
  ylab(expression(IR ~ (t/b)^2)) + theme(strip.background = element_blank(),
                                        strip.placement = "outside") +
  theme(legend.position = "none") +
  geom_errorbar(aes(x = Treatment, ymin = IR_mean - IR_SE, ymax = IR_mean + IR_SE),
               width = 0.4, colour = "black", alpha = 0.9, size = 0.02) +
  scale_fill_manual(values = c("grey90", "grey50")) +
  theme(axis.text.x = element_text(colour = "black", size = 13, angle = 0,
                                   margin = margin(t = 3))) +
  theme(axis.text.y = element_text(colour = "black", size = 13, angle = 0,
                                   margin = margin(r = 3))) +
  theme(axis.title.x = element_blank()) +
  theme(axis.title.y = element_text(size = 15, margin = margin(r = 3))) +
  theme(text = element_text(family = "Aerial", size = 15)) +
  geom_signif(comparisons = list(c("C", "D")), map_signif_level = TRUE,
              annotations = c("***"), y = 0.0125)
```

THEORETICAL HYDRAULIC CONDUCTIVITY (VESSELS)

(1) Prepare dataframe
First calculate hydraulically weighted vessel diameter (Dh), then theoretical hydraulic conductivity (Kth).
Calculations are with respect to a Dh and vessel frequency (VF) per m2 (Pfautsch et al., 2016).

(2) Control Kth calculation

```
HC_control <- Control_data %>%
  filter(Class == "Vessels") %>%
  group_by(Image) %>%
  summarise(Dh_Mean = (sum(Nucleus..Max.caliper^4/length(Class))^0.25) / 1e6,
            VF_m2 = length(Class)/1.46074377e-7)

HC_control$Kth <- ((HC_control$Dh_Mean^4 * pi) / (128 * 0.89e-9)) * HC_control$VF_m2 * 1000

HC_control$Treatment <- Control
```

(3) Droughted Kth calculation

```
HC_droughted <- Droughted_data %>%
  filter(Class == "Vessels") %>%
  group_by(Image) %>%
  summarise(Dh_Mean = (sum(Nucleus..Max.caliper^4/length(Class))^0.25) / 1e6,
            VF_m2 = length(Class)/1.46074377e-7)

HC_droughted$Kth <- ((HC_droughted$Dh_Mean^4 * pi) / (128 * 0.89e-9)) * HC_droughted$VF_m2 * 1000

HC_droughted$Treatment <- Droughted
```

(4) Calculating overall mean and SE for treatments (i.e. Control vs Droughted)

```r
HC_C_grouped <- HC_control %>%
  summarise(Kth_mean = mean(Kth),
            Kth_SE = sd(Kth)/sqrt(36))

HC_C_grouped$Treatment <- Control

HC_D_grouped <- HC_droughted %>%
  summarise(Kth_mean = mean(Kth),
            Kth_SE = sd(Kth)/sqrt(36))

HC_D_grouped$Treatment <- Droughted

HC_summary <- bind_rows(HC_C_grouped, HC_D_grouped, id = NULL)
```

(5) Checking normality
Shapiro-Wilks test

```r
shapiro.test(log(HC_control$Kth))
shapiro.test(log(HC_droughted$Kth))
```

(6) Significance / hypothesis testing

```r
t.test(log(HC_control$Kth),
       log(HC_droughted$Kth),
       paired = FALSE)
```

(7) Graphing
Kth Plot

```r
Kth_graph <- ggbarplot(HC_summary, x = "Treatment", y = "Kth_mean", fill = "Treatment") +
  ggtitle("Vessel") +
  theme(plot.title = element_text(hjust = 0.5, vjust = 2.5)) +
  ylab(expression(Kth ~ (kg ~ s^-1 ~ m^-1 ~ MPa^-1))) +
  theme(strip.background = element_blank(), strip.placement = "outside") +
  theme(legend.position = "none") +
  geom_errorbar(aes(x = Treatment, ymin = Kth_mean - Kth_SE, ymax = Kth_mean + Kth_SE),
              width = 0.4, colour = "black", alpha = 0.9, size = 0.02) +
  scale_fill_manual(values = c("grey90", "grey50")) +
  theme(axis.text.x = element_text(colour = "black", size = 13, angle = 0,
                                   margin = margin(t = 3))) +
  theme(axis.text.y = element_text(colour = "black", size = 13, angle = 0,
                                   margin = margin(r = 3))) +
  theme(axis.title.x = element_blank()) +
  theme(axis.title.y = element_text(size = 15, margin = margin(r = 3))) +
  theme(text = element_text(family = "Aerial", size = 15)) +
  geom_signif(comparisons = list(c("C", "D")), map_signif_level = TRUE,
              annotations = c("***"), y = 34.5)
```

(8) Creating grid plots

```
plot_grid(IR_graph, Kth_graph, labels = c('A', 'B'), label_size = 12, label_y = 0.99,
          ncol = 2)
```

FRACTIONAL CELL WALL AREA

(1) Calculating the fractional cell wall area percentage per slide / image (i.e. per tree sample)

```
FCWA_C <- Control_data %>%
  group_by(Image) %>%
  summarise(FCWA = (sum(CWA)/sum(Cell..Area)*100))

FCWA_D <- Droughted_data %>%
  group_by(Image) %>%
  summarise(FCWA = (sum(CWA)/sum(Cell..Area)*100))
```

(2) Calculating overall fractional cell wall area percentage and SE per treatment (i.e. Control vs Droughted)

```
FCWA_C_grouped <- FCWA_C %>%
  summarise(FCWA_mean = mean(FCWA),
            FCWA_SE = sd(FCWA)/sqrt(36))

FCWA_D_grouped <- FCWA_D %>%
  summarise(FCWA_mean = mean(FCWA),
            FCWA_SE = sd(FCWA)/sqrt(36))
```

(3) Checking normality
Shapiro-Wilks test

```
shapiro.test(FCWA_C$FCWA)
shapiro.test(FCWA_D$FCWA)
```

(4) Significance / hypothesis testing

```
t.test(FCWA_C$FCWA,
       FCWA_D$FCWA,
       paired = FALSE)
```

LIGNIN CONTENT PER UNIT AREA

(1) Calculating the lignin proportional area per slide / image (i.e. per tree sample)

```
lignin_C <- Control_data %>%
  group_by(Image) %>%
  summarise(LWA = (sum(CWA*0.3009)/sum(Cell..Area)*100))

lignin_D <- Droughted_data %>%
  group_by(Image) %>%
  summarise(LWA = (sum(CWA*0.2910)/sum(Cell..Area)*100))
```

(2) Calculating the overall lignin proportional area percentage and SE per treatment (i.e. Droughted vs Control)

```
lignin_C_grouped <- lignin_C %>%
  summarise(LWA_mean = mean(LWA),
            LWA_SE = sd(LWA)/sqrt(36))

lignin_D_grouped <- lignin_D %>%
  summarise(LWA_mean = mean(LWA),
            LWA_SE = sd(LWA)/sqrt(36))
```

(3) Checking normality
Shapiro-Wilks test

```
shapiro.test(lignin_C$LWA)
shapiro.test(lignin_D$LWA)
```

(4) Significance / hypothesis testing

```
t.test(lignin_C$LWA,
       lignin_D$LWA,
       paired = FALSE)
```
