# Supplementary material for: Xylem cell size regulation is a key adaptive response to water deficit in Eucalyptus grandis
Source: Tree Physiol. 2024 Jun 18;44(7):tpae068. doi: 10.1093/treephys/tpae068 (PMC11247191; doi:10.1093/treephys/tpae068)
Supplement: Code_transcriptomics_tpae068 [file code_transcriptomics_tpae068.pdf]

Differential gene expression and functional analysis in *Eucalyptus grandis* exposed to water deficit

Rafael Keret

2024-02-13

All code and dataframes are available as R projects on GitHub:

https://github.com/Rafael-Keret/Eucalyptus_transcriptomic_analysis

DESeq2 ANALYSIS OF COUNTS MATRIX

LOAD PACKAGES

```
library("DESeq2")
library("dplyr")
library("ggrepel")
library("stringr")
```

PREPARE FEATURECOUNTS DATA FILE (i.e. counts matrix)

(1) Import data and rename columns

```
fc_output <- read.csv("./Data/input/FC_NCBI_GTF", head = TRUE, sep = "\t", skip = 1)
fc_output <- fc_output[, c(1, 7:14)]
colnames(fc_output) <- c("qseqid", "C1", "C2", "C3", "C4", "D1", "D2", "D3", "D4")
fc_output$qseqid <- gsub("LOC", "", fc_output$qseqid)

length(unique(fc_output$qseqid))

fc_output <- data.frame(fc_output, row.names = 1)
```

(2) Set factor or treatment level(s) from pheno data file (i.e. what you are testing against in your experiment)

```
DESeq_pheno_data <- read.csv("./Data/input/DESeq_pheno_data.csv", head = TRUE, sep = ",",
                             row.names = 1)

DESeq_pheno_data$treatment <- factor(DESeq_pheno_data$treatment)
```

(3) Create a DESeq object with read counts and pheno data
If you have multiple factors add these to "design =" below.

Then you need to specify your factors in the order of increasing importance.
For instance, if you have species and drought, but you are more interested in the overall impact of drought then specify species as your first factor, and drought as your second (i.e. "design = ~ species + drought").

```
dds <- DESeqDataSetFromMatrix(countData = fc_output, colData = DESeq_pheno_data,
                              design = ~treatment)
```

(4) Specify the reference for treatment factor
Set control (well-watered) as a reference (i.e. droughted divided by control to calculate fold change). The one that is specified first becomes the reference which to compare to.

```
dds$treatment <- factor(dds$treatment, levels = c("C", "D"))
```

(5) Filter for genes that have read counts (counts per million) greater than or equal to 5
Soewarto et.al, 2019 M&M provide a good explanation of this.

```
keep <- rowSums(counts(dds)) >= 5
dds <- dds[keep,]
```

STATISTICS

(1) Identify differentially expressed genes (DEGs)
The DESeq function automatically normalizes for sequencing depth and composition before performing DEA.
The method is called "median of ratios".

```
dds <- DESeq(dds)
deseq_result <- results(dds)
deseq_result
```

(2) Look at a summary of our results

```
summary(deseq_result)  # This summarizes the data based on a p-value < 0.1

deseq_result_0.01 <- results(dds, alpha = 0.01) # can change it to a lower p-value = 0.01
summary(deseq_result_0.01)
```

(3) Change deseq_result into dataframe, and order on p-value

```
deseq_result <- as.data.frame(deseq_result)
deseq_result <- deseq_result[order(deseq_result$pvalue),]
write.csv(deseq_result, "./Data/deseq_result.csv")
```

(4) Create background gene list to retrieve FASTA sequences or gene models from NCBI

```
gene_list <- rownames(deseq_result)

write.table(gene_list, "./Data/ncbi_gene_list.txt", sep="\t", row.names = FALSE,
            quote = FALSE, col.names = FALSE)
```

(5) Some queries that can be used
Check if a specific gene is DE, down or up regulated?
Pick an arbitrary gene for this example (LOC104456901), and call it from deseq_result.

```
deseq_result["104456901",]
```

(6) Extract the most differentially expressed genes
Select genes with a q-value (i.e. adjusted p-value) < 0.05 and a |log2FoldChange| > 1 (i.e. 2x expression).

```
deseq_filtered <- deseq_result %>% filter(deseq_result$padj < 0.05)
deseq_filtered <- deseq_filtered %>% filter(abs(deseq_filtered$log2FoldChange) > 1)

write.csv(deseq_filtered, "./Data/deseq_filtered.csv")
```

(7) Create a differentially expressed gene list

```
DEG_list <- rownames(deseq_filtered)

write.table(DEG_list, "./Data/ncbi_DEG_list.txt", sep="\t", row.names = FALSE,
            quote = FALSE, col.names = FALSE)
```

(8) Create a normalized dataframe based on library depth and composition

```
normalized_counts <- counts(dds, normalized = TRUE)
normalized_counts <- as.data.frame(normalized_counts)
write.csv(normalized_counts, "./Data/normalized_counts.csv")
```

DISPERSION PLOT

```
plotDispEsts(dds)
```

PCA PLOT

(1) Variance stabilizing transformation

```
vsd <- vst(dds, blind = FALSE)
```

(2) Use transformed values to generate PCA plot

```
plotPCA(vsd, intgroup = "treatment")
```

HEATMAPS

Heatmap of sample-to-sample distance matrix (with clustering) based on normalised counts.

To determine which samples as a whole are most related to each other, based on gene expression patterns.

(1) Load packages

```
library("pheatmap")
library("RColorBrewer")
```

(2) Create distance matrix

```
sampleDists <- dist(t(assay(vsd)))
sampleDistMatrix <- as.matrix(sampleDists)
```

(3) Colour scheme

```
colours <- colorRampPalette(rev(brewer.pal(9, "Blues")))(255)
```

(4) Plot

```
pheatmap(sampleDistMatrix, clustering_distance_rows = sampleDists,
         clustering_distance_cols = sampleDists, col = colours)
```

(5) Heatmap of log2 transformed normalized counts (i.e. library size & comp)
Top 30 differentially expressed genes.

```
top_deg <- deseq_result[order(deseq_result$padj), ][1:30, ]
top_deg <- row.names(top_deg)

rld <- rlog(dds, blind = FALSE)

treatment_col = list(treatment = c(C = "skyblue", D = "orange2"))

pheatmap(assay(rld)[top_deg, ], cluster_rows = TRUE, show_rownames = TRUE,
         cluster_cols = TRUE, annotation_col = DESeq_pheno_data,
         annotation_colors = treatment_col,
         color = colorRampPalette(c("black", "green", "red"))(50))
```

(6) Heatmap of Z-scores
Top 30 differentially expressed genes.

```
cal_z_score <- function(x) {(x - mean(x)) / sd(x)}

z_score_all <- t(apply(normalized_counts, 1, cal_z_score))

z_score_subset <- z_score_all[top_deg,]

pheatmap(z_score_subset, cluster_rows = TRUE, show_rownames = TRUE, cluster_cols = TRUE,
         annotation_col = DESeq_pheno_data, annotation_colors = treatment_col,
         color=colorRampPalette(c("green", "black", "red"))(50))
```

MA PLOT

(1) Load package and plot raw untransformed MA plot

```
library("apeglm")

plotMA(dds, ylim = c(-8, 8))
```

Remove noise of low counts with high dispersion (background noise).
This finds instances where the log fold change was exaggerated and shrinks them down.
Previously, we got higher dispersions (i.e. variances) at a lower "mean of normalised counts".
So the dispersion plot tells us that the fold change will be exaggerated at lower mean counts because the variance was higher in genes of lower expression.

(2) Transform via "apeglm" model

```
res_apeglm_MA <- lfcShrink(dds, coef = "treatment_D_vs_C", type = "apeglm")
plotMA(res_apeglm_MA, ylim = c(-5, 5))
```

(3) Transform via "normal" model

```
res_normal_MA <- lfcShrink(dds, coef = "treatment_D_vs_C", type = "normal")
plotMA(res_normal_MA, ylim = c(-5, 5))
```

VOLCANO PLOT APEGLM

(1) Load packages

```
library("data.table")
library("ggplot2")
library("ggrepel")
library("dplyr")
```

(2) Change the normalized res_apeglm_MA data, to a dataframe

```
resLFC_ape <- as.data.frame(res_apeglm_MA) %>% setDT(keep.rownames = "gene_id")
```

(3) Label genes as up, down or not regulated

```
resLFC_ape$diffexpressed <- "NO"
resLFC_ape$diffexpressed[resLFC_ape$log2FoldChange > 1 & resLFC_ape$padj < 0.05] <- "UP"
resLFC_ape$diffexpressed[resLFC_ape$log2FoldChange < -1 & resLFC_ape$padj < 0.05] <- "DOWN"
```

(4) Create a labeling column for genes of interest

```
resLFC_ape$delabel <- NA
```

(5) Set the threshold p-value at which labels should be included

```
threshold <- head(arrange(resLFC_ape, pvalue), 10)$pvalue[10]
```

(6) Add gene id labels based on values that are lower than or equal to the threshold
Start with a query and end with what we want to perform should the query be fulfilled.

```
resLFC_ape$delabel[resLFC_ape$pvalue <= threshold & !is.na(resLFC_ape$pvalue)] <-
  (resLFC_ape$gene_id[resLFC_ape$pvalue <= threshold & !is.na(resLFC_ape$pvalue)])
```

(7) Plot volcano
Noise reduced data.

```
ggplot(data = resLFC_ape,
       aes(x = log2FoldChange, y = -log10(pvalue), col = diffexpressed, label = delabel)) +
  geom_point() + theme_minimal() + geom_text_repel() +
  scale_colour_manual(values = c("green3", "grey", "red3")) +
  theme(text = element_text(size = 12)) +
  geom_vline(xintercept = c(-1, 1), col = "black", linetype = 2) +
  geom_hline(yintercept = -log10(0.01), col = "black", linetype = 2)
```

VOLCANO PLOT NORMAL

(1) Change the normalized res_normal_MA data, to a dataframe

```
resLFC_norm <- as.data.frame(res_normal_MA) %>% setDT(keep.rownames = "gene_id")
```

(2) Label genes as up, down or not regulated

```
resLFC_norm$diffexpressed <- "NO"
resLFC_norm$diffexpressed[resLFC_norm$log2FoldChange > 1 & resLFC_norm$padj < 0.05] <- "UP"
resLFC_norm$diffexpressed[resLFC_norm$log2FoldChange < -1 & resLFC_norm$padj < 0.05] <- "DOWN"
```

(3) Label genes of interest

```
resLFC_norm$delabel <- NA
```

(4) Set the threshold for p-value at which labels should be included

```
threshold <- head(arrange(resLFC_norm, pvalue), 10)$pvalue[10]
```

(5) Add gene id labels based on values that are lower than or equal to the threshold
Start with a query and end with what we want to perform should the query be fulfilled.

```
resLFC_norm$delabel[resLFC_norm$pvalue <= threshold & !is.na(resLFC_norm$pvalue)] <-
  (resLFC_norm$gene_id[resLFC_norm$pvalue <= threshold & !is.na(resLFC_norm$pvalue)])
```

(6) Plot volcano
Noise reduced data.

```
ggplot(data = resLFC_norm,
       aes(x = log2FoldChange, y = -log10(pvalue), col = diffexpressed, label = delabel)) +
  geom_point() + theme_minimal() + geom_text_repel() +
  scale_colour_manual(values = c("green3", "grey", "red3")) +
  theme(text = element_text(size = 12)) +
  geom_vline(xintercept = c(-1, 1), col = "black", linetype = 2) +
  geom_hline(yintercept = -log10(0.01), col = "black", linetype = 2)
```

RAW VOLCANO PLOT

(1) Convert to dataframe and change first column name

```
deseq_result <- as.data.frame(deseq_result) %>% setDT(keep.rownames = "gene_id")
```

(2) Label genes as up, down or not regulated

```
deseq_result$diffexpressed <- "NO"
deseq_result$diffexpressed[deseq_result$log2FoldChange > 1 & deseq_result$padj < 0.05] <- "UP"
deseq_result$diffexpressed[deseq_result$log2FoldChange < -1 & deseq_result$padj < 0.05] <- "DOWN"

deseq_result$delabel <- NA
```

(3) Check and set the threshold for p-value and fold change at which labels should be included

```
threshold <- head(arrange(deseq_result, pvalue), 10)$pvalue[10]
```

(4) Add gene labels based on values that are lower than or equal to the threshold
Start with a query and end with what we want to perform should the query be fulfilled.

```
deseq_result$delabel[deseq_result$pvalue <= threshold & !is.na(deseq_result$pvalue)] <-
  (deseq_result$gene_id[deseq_result$pvalue <= threshold & !is.na(deseq_result$pvalue)])
```

(5) Plot volcano

```
ggplot(data = deseq_result,
       aes(x = log2FoldChange, y = -log10(pvalue), col = diffexpressed, label = delabel)) +
  geom_point() + theme_minimal() + geom_text_repel() +
  scale_colour_manual(values = c("green3", "grey", "red3")) +
  theme(text = element_text(size = 12)) +
  geom_vline(xintercept = c(-1, 1), col = "black", linetype = 2) +
  geom_hline(yintercept = -log10(0.01), col = "black", linetype = 2)
```

ANNOTATION OF EUCALYPTUS GRANDIS ENTREZ IDS

INSTALL AND LOAD PACKAGES

```
BiocManager::install("AnnotationDbi")
BiocManager::install("org.At.tair.db")
BiocManager::install("GO.db")
BiocManager::install("clusterProfiler")
BiocManager::install("limma")

library("AnnotationDbi")
library("org.At.tair.db")
library("GO.db")
library("dplyr")
library("stringi")
library("clusterProfiler")
library("splitstackshape")
```

IMPORT AND PREPARE DATA

(1) BLASTX results table

```
blast <- read.table("./Data/input/blastx_orthologs_ncbi.txt", sep="\t", header = FALSE)
colnames(blast)  <- c("qseqid", "sseqid", "pident", "length", "mismatch", "evalue", "bitscore")
blast$sseqid <- gsub("\\..*", "", blast$sseqid)
```

(2) Select the top A.thaliana TAIR ID hit for each unique E.grandis ENTREZ gene ID

```
blast <- blast %>%
  group_by(qseqid) %>%
  slice_min(evalue) %>%
  slice_max(bitscore) %>%
  slice_max(length) %>%
  slice_min(mismatch) %>%
  ungroup

length(unique(blast$qseqid))
```

(3) Retain unique E. grandis ENTREZ IDs with a corresponding TAIR ortholog

```
blast <- blast[which(!duplicated(blast$qseqid)), ]
```

(4) Rename chromosomal regions to ENTREZ ID

```
blast$qseqid <-
  stri_replace_all_regex(blast$qseqid,
                        pattern=c("NC_014570.1:c139556-136747", "NC_052613.1:3272908-3275532",
                                  "NC_052613.1:38624984-38627143", "NC_052613.1:c55328170-55325438",
                                  "NC_052614.1:11229172-11231700", "NC_052616.1:c28054277-28051374",
                                  "NC_052617.1:c20483764-20479776", "NC_052617.1:c34031899-34029393",
                                  "NC_052617.1:c40716758-40710803", "NC_052618.1:51481974-51505466",
                                  "NC_052620.1:c30982539-30978927", "NC_052620.1:c35825863-35822327",
                                  "NC_052621.1:753837-759217", "NC_052622.1:32327277-32331798"),
```

```
                          replacement=c("9845767", "104418091", "104432674", "104433897", "104436537",
                                        "104441567", "104448849", "104449214", "104449759", "104454102",
                                        "104444074", "104419574", "104420918", "104426035"),
                          vectorize=FALSE)
```

(5) Export final BLASTX results table

```
write.table(blast, "./Data/output/blastx_top_hits.txt", sep="\t", row.names = FALSE,
            quote = FALSE, col.names = TRUE)
```

CLUSTERPROFILER GO ANNOTATION

(1) Select levels 1 to 10 for GO term searches, and only select biological processes ("BP")

```
ggo <- groupGO(gene     = blast$sseqid,
               OrgDb    = org.At.tair.db,
               ont      = "BP",
               level    = c(1:10),
               readable = FALSE,
               keyType = "TAIR")

ggo_DF <- as.data.frame(ggo)
```

According to the "ggo_DF" table ("Biological process") 13078 / 13577 TAIR IDs have been annotated.

(2) Split the individual TAIR IDs in the GeneID column into individual rows

```
GO_At <- concat.split.multiple(ggo_DF, "geneID", seps="/", "long")
colnames(GO_At)[5] <- "sseqid"
GO_At <- distinct(GO_At)

write.table(GO_At, "./Data/output/GO_At.txt", sep="\t", row.names = FALSE,
            quote = FALSE, col.names = TRUE)
```

GENE SYMBOL, DESCRIPTION AND ONTOLOGY

(1) Load biomaRt package then retrieve A.thaliana gene symbol (TAIR) and function

```
library("biomaRt")

tair_mart <- useMart(biomart = "plants_mart",
                     host = "plants.ensembl.org", dataset = "athaliana_eg_gene")

At_symbols <- getBM( values = blast$sseqid,
                     mart = tair_mart,
                     attributes = c("ensembl_gene_id", "entrezgene_id",
                                    "description", "external_gene_name"),
                     filters = "ensembl_gene_id")

colnames(At_symbols)[c(1, 3)] <- c("sseqid", "function")
```

(2) Export gene symbol and function file

```
write.csv(At_symbols, "./Data/output/gene_symbols_function.csv", row.names = FALSE)
```

FUNCTIONAL ANALYSIS OF DIFFERENTIALLY EXPRESSED GENES

REFERENCES

(1) ClusterProfiler
Wu T, Hu E, Xu S, Chen M, Guo P, Dai Z, Feng T, Zhou L, Tang W, Zhan L, Fu x, Liu S, Bo X, Yu G (2021). "clusterProfiler 4.0: A universal enrichment tool for interpreting omics data." The Innovation, 2(3), 100141. doi: 10.1016/j.xinn.2021.100141.

(2) Arabidopsis thaliana database
Carlson M (2019). org.At.tair.db: Genome wide annotation for Arabidopsis. R package version 3.8.2.

INSTALL AND LOAD PACKAGES

```
if (!require("BiocManager", quietly = TRUE))
  install.packages("BiocManager")

BiocManager::install("clusterProfiler")
BiocManager::install("pathview")
BiocManager::install("Rgraphviz")
BiocManager::install("enrichplot")

library("clusterProfiler")
library("enrichplot")
library("ggplot2")
library("dplyr")
library("tidyverse")
library("stringi")
library("pheatmap")
```

IMPORT AND PREPARE DATA

(1) Import DESeq2 output data

```
deseq_out <- read.csv("./Data/input/deseq_result.csv", head = TRUE)
colnames(deseq_out)[1]  <- "qseqid"
```

(2) Rename gene symbols to ENTREZ ID

```
deseq_out$qseqid <-
  stri_replace_all_regex(deseq_out$qseqid,
                         pattern=c("EucgrC_r007", "ISU1", "TUB2", "TUB5", "MIOX", "TUB4",
                                   "TUB3", "TUA1","UGP", "NFU4", "EGM2", "WOX13.2",
                                   "UXS1", "EGM3"),
                         replacement=c("9845767", "104418091", "104432674", "104433897", "104436537",
```

```
                                           "104441567", "104448849", "104449214", "104449759", "104454102",
                                           "104444074", "104419574", "104420918", "104426035"),
                              vectorize=FALSE)

length(unique(deseq_out$qseqid))
```

(3) Import BLASTX orthologs (i.e. Arabidopsis thaliana orthologs)

```
blast <- read.table("./Data/input/blastx_top_hits.txt", sep="\t", header = TRUE)
blast$qseqid <- as.character(blast$qseqid)
```

(4) Left join by blast, to retain all ENTREZ ID's that have a corresponding A.thaliana ortholog
Only export "qseqid" and "log2FoldChange" that are relevant to MapMan software.

```
deseq_orth <- left_join(blast, deseq_out, by = "qseqid")
deseq_orth <- deseq_orth[which(!duplicated(deseq_orth$qseqid)), ]
length(unique(deseq_orth$qseqid))

write.csv(deseq_orth, file = "./Data/output/deseq_orth.csv", row.names = FALSE)
write.table(na.omit(deseq_orth[, c("qseqid", "log2FoldChange")]),
          file = "./Data/output/deseq_orth_mapman.txt", row.names = FALSE, sep = "\t")
```

(5) Create a filtered dataframe for investigation of xylogenesis related genes
Select differentially expressed genes with a "padj < 0.05", and a "|L2FC| > 1.0" for data mining.

```
deseq_filtered <- deseq_orth %>% filter(deseq_orth$padj < 0.05)
deseq_filtered <- deseq_filtered %>% filter(abs(deseq_filtered$log2FoldChange) > 1)
```

(6) Create gene ontology (GO) ID and terms table

```
GO <- read.table("./Data/input/GO_At.txt", sep="\t", header = TRUE, quote = "")
GO <- GO[, c(1,2,5)]
```

(7) Left join to assign GO terms to E. grandis ENTREZ IDs

```
GO_assignment <- left_join(deseq_orth, GO, by = "sseqid", relationship = "many-to-many")

length(unique(GO_assignment$qseqid))
```

CLUSTERPROFILER GSEA

(1) Create term2gene and term2name tables

```
term2gene <- GO_assignment[, c(14, 1)] %>% select(ID:qseqid)

term2name <- GO_assignment[, c(14, 15)]

length(unique(term2gene$qseqid))
```

(2) Create a ranked list for GSEA, ClusterProfiler uses Log2foldchange as a ranking metric POSSILBE ALTERNATIVE RANKING METRIC: Deseq_orth <- mutate(Deseq_orth, rank = sign(log2FoldChange) * -log10(padj)).

(3) Extract Log2FoldChange values and corresponding query sequence ID ("qseqid")

```
all_genes <- deseq_orth$log2FoldChange

names(all_genes) <- deseq_orth$qseqid
```

(4) Remove NAs

```
genes <- na.omit(all_genes)
```

(5) Sort the ranked values in decreasing order

```
genes <- sort(genes, decreasing = TRUE)
```

(6) Gene Set Enrichment Analysis
Perform gene set enrichment analysis using GO terms.
The software (warning message) recommended to NOT set the permutation level.
Remember to set the seed to TRUE for consistent results as this is a randomization method.

```
gseGO <- GSEA(genes,
             minGSSize = 50,
             maxGSSize = 480,
             pvalueCutoff = 0.05,
             pAdjustMethod = "BH",
             TERM2GENE = term2gene,
             TERM2NAME = term2name,
             verbose = TRUE,
             seed = TRUE,
             eps = 0)

gse_DF <- as.data.frame(gseGO)
write.csv(gse_DF, file = "./Data/output/Table_S7_gsea.csv", row.names = FALSE)
```

HISTOGRAM OF RELEVANT / NON-REDUNDANT ENRICHED CATEGORIES

(1) Load packages

```
library("forcats")
require(DOSE)
```

(2) Histogram of categories of interest
Choose biological processes (BP) from the gene set enrichment analysis (GSEA) list that are relevant to the research questions.
Additionally remove potentially redundant categories.

```
selected_ont <- c(31, 107, 43, 20, 19, 133, 191, 184, 192, 168, 139,
                  126, 134, 199, 200, 37, 177, 25, 153, 81, 79, 76, 9, 1,
                  175, 12, 173)

gseGO_histogram <- data.frame(Description = gseGO$Description[selected_ont],
                              ID = gseGO$ID[selected_ont],
                              NES = gseGO$NES[selected_ont],
                              qvalue = gseGO$qvalue[selected_ont])
```

(3) Function to assign a generalized process to a BP based on partial matches

```
assign_process <- function(x) {
  query_terms <- c("GO:0006260", "GO:0044786", "GO:0009826", "GO:0007018", "GO:0000910",
                   "GO:0016051", "GO:1901659", "GO:1901361", "GO:0072329", "GO:0015980",
                   "GO:0009698", "GO:0009813", "GO:0016102", "GO:0016114", "GO:0016144",
                   "GO:0045491", "GO:0010411", "GO:0010410", "GO:0009825", "GO:0070592",
                   "GO:0042545", "GO:0009664", "GO:0000302", "GO:0009408", "GO:0034050",
                   "GO:0042542", "GO:0009626")
  set_terms <- c("Development", "Development", "Development", "Development", "Development",
                 "Primary", "Primary", "Primary", "Primary", "Primary",
                 "Secondary", "Secondary", "Secondary", "Secondary", "Secondary",
                 "Cell wall", "Cell wall", "Cell wall", "Cell wall", "Cell wall",
                 "Cell wall", "Cell wall", "Stress", "Stress", "Stress", "Stress",
                 "Stress")

  for (i in seq_along(query_terms)) {
    if (grepl(query_terms[i], x, fixed = TRUE)) {
      return(set_terms[i])
    }
  }
  return(NA)  # If no partial match is found, return NA or any other default value
}

gseGO_histogram$process <- sapply(gseGO_histogram$ID, assign_process)
```

(4) Arrange based on process and NES column, then rename “qvalue”

```
gseGO_histogram <- arrange(gseGO_histogram, process, NES)
gseGO_histogram <- rename(gseGO_histogram, p.adj = qvalue)
```

(5) Select order of bars, and plot

```
desired_order <- gseGO_histogram$Description

histo <- ggplot(gseGO_histogram, aes(NES, factor(Description, levels = desired_order),
                                     fill = p.adj)) +
  geom_col() +
  scale_fill_gradientn(colours = c("#b3eebe", "#46bac2", "#371ea3"),
                       guide = guide_colorbar(reverse = TRUE)) +
  theme_minimal() +
  labs(x = "NES", y = NULL) +
```

```r
  scale_y_discrete(labels = function(x) str_wrap(x, width = 90), expand = c(0.005, 0)) +
  theme(axis.text.y = element_text(size = 15), axis.title.x = element_text(size = 15),
        axis.text.x = element_text(size = 15)) +
  theme(plot.margin = unit(c(1, 0, 3.0, 3.0), "lines")) +
  theme( legend.text = element_text(size = 15), legend.title = element_text(size = 15),
         legend.key.size = unit(1, "lines"))
```

ENRICHMENT NETWORK MAP OF SIGNIFICANTLY ENRICHED ONTOLOGIES

(1) Enrichment map showing category interactions, functional association or shared genes
To remove redundant GO terms.
node_label = "category" or "none".

```r
gseGO_2 <- pairwise_termsim(gseGO)

set.seed(24)
emapp <- emapplot(gseGO_2, showCategory = gseGO_2$Description[c(1:200)], color = "NES",
         cex.params = list(category_node = 0.5, category_label = 0.1,
                           line = 0.3, label_group = 0.5), node_label = "category",
         cluster.params = list(cluster = FALSE, legend = TRUE, n = 2,
                               label_style = "shadowtext", label_words_n = 1,
                               label_format = 5, repel = TRUE, group_legend = TRUE,
                               method = stats::kmeans),
         edge.params = list(show = TRUE, min = 0.08))
```

(2) Join histogram and network plot

```r
cowplot::plot_grid(emapp, histo, ncol = 1, labels = LETTERS[1:2],
                   rel_widths = c(.8, .8, 1.2), label_size = 22)
```

EXTRACT AND LABEL DIFFERENTIALLY EXPRESSED GENES

(1) Read in gene symbol and functions table

```r
gene_symbols_function <- read.csv("./Data/input/gene_symbols_function.csv", head = TRUE)
```

(2) Left join to add gene names and descriptions to filtered E. grandis ENTREZ IDs

```r
gene_symbol <- left_join(deseq_filtered, gene_symbols_function, by = "sseqid",
                         relationship = "many-to-many")

gene_symbol <- select(gene_symbol, c(1, 2, 9, 10, 13, 15, 16))

gene_symbol <- distinct(gene_symbol)

length(unique(gene_symbol$qseqid))

write.csv(gene_symbol, "./Data/output/Table_S8_degs.csv", row.names = FALSE)
```

QUERY FILTERING OF DEGs

(1) Import MapMan classification list to help identify genes relevant to xylogenesis

```
mapman_classifications <- read.delim("./Data/input/MapMan_classifications.txt", header = TRUE)
mapman_classifications <- rename(mapman_classifications, qseqid = id, L2FC = deseq_orth_mapman.txt)
mapman_classifications$qseqid <- as.character(mapman_classifications$qseqid)
mapman_classifications <- left_join(deseq_filtered, mapman_classifications, by = "qseqid") %>%
  select(BinCode, BinName, qseqid, type, description, L2FC)
```

(2) Identify genes relevant to wood formation or xylogenesis
Genes related to cellular expansion and cell wall component biosynthesis.

```
xylem_development_ID <- c("104433767", "104433819", "104433834", "104433865", "104433885",
                          "104454356", "104442333", "104436371", "104444630", "104444640",
                          "104444649", "104449794", "104452753", "104452752", "104421716",
                          "104421040", "104427839", "104421714", "104450069", "104448602",
                          "104434058", "104433548", "104443623", "104443626", "104428622",
                          "104432727", "104432728", "104424356", "104440602", "104441874",
                          "104419526", "104443687", "104414336", "104456622", "104453686",
                          "104426781", "104424340", "104424341", "104456165", "108953967",
                          "104454145", "104425949", "104417282", "104414384", "104454440",
                          "104430693", "104419915", "104420951", "104449520", "104446186",
                          "104423212", "104449672", "104420130", "104436967", "104450842",
                          "104450844", "104454116", "104442048", "104441676", "104441677",
                          "104420942", "104415214", "104432862", "104421673", "104441055",
                          "104416110", "104425811", "104449510", "104435787", "104457357",
                          "104415154", "104450362")
```

(3) Extract relevant ENTREZ IDs from differentially expressed gene set

```
xylogenesis_query <- gene_symbol %>%
  filter(grepl(paste(xylem_development_ID, collapse = "|"), qseqid, ignore.case = TRUE),
         abs(log2FoldChange) >= 1.0, padj <= 0.05)
```

(4) Replace TAIR ID, with gene symbol, and create gene id/symbol column

```
xylogenesis_query$external_gene_name <- stri_replace_all_regex(xylogenesis_query$external_gene_name,
                                                 pattern=c("AT1G02460", "AT1G04680", "AT1G14890",
                                                           "AT1G55770", "AT2G38150", "AT3G18180",
                                                           "AT3G24130", "AT3G50990", "AT3G53190",
                                                           "AT3G55700", "AT3G57380", "AT3G59850",
                                                           "AT4G19420", "AT4G24780", "AT4G30380",
                                                           "AT5G19730", "AT5G39580", "AT5G62360",
                                                           "AT1G62660"),
                                                 replacement=c("PLL", "PLL26", "PMEI", "PMEI",
                                                               "1,4-GTF", "GT61", "PME29", "PRX36",
                                                               "PLL17", "UGT76F1", "GT61", "PLL",
                                                               "PAE", "PLL19", "EXLB2", "PME53",
                                                               "PRX62", "PMEI13", "VI1"),
                                                 vectorize=FALSE)
```

```
xylogenesis_query$ID_symbol <-
  paste(xylogenesis_query$qseqid, "(", xylogenesis_query$external_gene_name, ")", sep="")
```

(5) Arrange based on xylem_development_ID

```
xylogenesis_query <- xylogenesis_query[order(match(xylogenesis_query$qseqid, xylem_development_ID)), ]
xylogenesis_query <- rename(xylogenesis_query, p.adj = padj)
```

(6) Break data up for 2 plots

```
xylogenesis_query_1 <- xylogenesis_query[1:36, ]
xylogenesis_query_2 <- xylogenesis_query[37:72, ]
```

(7) Invert xylogenesis_query_2 for plotting purposes

```
xylogenesis_query_2 <- xylogenesis_query_2[rev(seq_len(nrow(xylogenesis_query_2))), ]
```

(8) Plot data

```
xylogeneis_order <- xylogenesis_query$ID_symbol

x1 <- ggplot(xylogenesis_query_1,
             aes(x = log2FoldChange, y = factor(ID_symbol, levels = xylogeneis_order),
                 fill = p.adj)) +
  geom_bar(stat = "identity", orientation = "y") +
  scale_fill_gradientn(colours=c("#b3eebe", "#46bac2", "#371ea3")) +
  labs(x = expression("Log"[2]~"fold change"), y = NULL, fill = "p.adj") + theme_minimal() +
  geom_errorbar(aes(x = log2FoldChange,
                    xmin = log2FoldChange - lfcSE, xmax = log2FoldChange + lfcSE),
                width = 0.4, colour = "black", alpha = 0.9, size = 0.02) +
  theme(panel.grid.major.x = element_line(color = "grey90", size = 0.5),
        panel.grid.major.y = element_line(color = "grey90", size = 0.5)) +
  theme(plot.margin = unit(c(2, 0, 0, 1.8), "lines")) +
  theme(axis.text.y = element_text(size = 18), axis.title.x = element_text(size = 18),
        axis.text.x = element_text(size = 18)) +
  theme( legend.text = element_text(size = 12), legend.title = element_text(size = 12),
         legend.key.size = unit(1.0, "lines")) +
  scale_y_discrete(limits=rev) + scale_x_continuous(breaks = seq(-6, 6, by = 3))

x2 <- ggplot(xylogenesis_query_2,
             aes(x = log2FoldChange, y = factor(ID_symbol, levels = xylogeneis_order),
                 fill = p.adj)) +
  geom_bar(stat = "identity", orientation = "y") +
  scale_fill_gradientn(colours=c("#b3eebe", "#46bac2", "#371ea3")) +
  labs(x = expression("Log"[2]~"fold change"), y = NULL, fill = "p.adj") + theme_minimal() +
  geom_errorbar(aes(x = log2FoldChange,
                    xmin = log2FoldChange - lfcSE, xmax = log2FoldChange + lfcSE),
                width = 0.4, colour = "black", alpha = 0.9, size = 0.02) +
  theme(panel.grid.major.x = element_line(color = "grey90", size = 0.5),
        panel.grid.major.y = element_line(color = "grey90", size = 0.5)) +
```

```r
  theme(plot.margin = unit(c(2, 0, 0, 1.8), "lines")) +
  theme(axis.text.y = element_text(size = 18), axis.title.x = element_text(size = 18),
        axis.text.x = element_text(size = 18)) +
  theme( legend.text = element_text(size = 12), legend.title = element_text(size = 12),
         legend.key.size = unit(1.0, "lines")) +
  scale_y_discrete(limits=rev) + scale_x_continuous(breaks = seq(-6, 6, by = 3))

cowplot::plot_grid(x1, x2, ncol = 2, labels = LETTERS[1:2], rel_widths = c(1, 1, 1.2),
                   label_size = 26)
```

TRANSCRIPTION FACTORS GOVERNING XYLEM DEVELOPMENT

(1) Enter TF ENTREZ IDs relevant to wood formation

```r
TF_ID <- c("104419933", "104448414", "104418025", "104450926", "104452408", "104436535",
           "104424951", "104433270", "104436346", "104442190", "104450012", "104453968",
           "104448388", "104426573", "104414409", "104415262", "104434101", "104426233",
           "104437240", "104438144", "104418936", "104453423", "104443670", "104424339",
           "104442593", "104417786", "104433840", "104425859", "104424612", "104431568",
           "104432590", "104454661", "104449656", "104455407", "104419371")
```

(2) Extract relevant ENTREZ IDs from differentially expressed gene set

```r
TF <- gene_symbol %>%
  filter(grepl(paste(TF_ID, collapse = "|"), qseqid, ignore.case = TRUE),
         abs(log2FoldChange) >= 1.0, padj <= 0.05)
```

(3) Replace TAIR ID, with gene symbol, and create gene id/symbol column

```r
TF$external_gene_name <- stri_replace_all_regex(TF$external_gene_name,
                                                pattern=c("MYB102", "MYB305", "MYB57", "ATAF1"),
                                                replacement=c("MYB41", "MYB13", "MYB4", "NAC2"),
                                                vectorize=FALSE)

TF$ID_symbol <- paste(TF$qseqid, "(", TF$external_gene_name, ";",
                      round(TF$log2FoldChange, 2), ")", sep = " ")
```

(4) Order TFs based on common function

```r
TF <- subset(TF, grepl(paste(TF_ID, collapse = "|"),
                       qseqid))[match(TF_ID,
                                      subset(TF, grepl(paste(TF_ID, collapse = "|"),
                                                       qseqid))$qseqid), ]
```

(5) Create matrix for heatmap

```r
TF_matrix <- matrix(TF$log2FoldChange, nrow = nrow(TF))
```

(6) Set the row and column names for the heatmap

```r
rownames(TF_matrix) <- TF$ID_symbol
colnames(TF_matrix) <- "MYB and NAC TFs"
```

(7) Specify breaks for colour palette, to set zero as "white"

```r
breaks_TF <- c(seq(min(TF_matrix), -1e-10, length.out = 50), 0,
               seq(1e-10, max(TF_matrix), length.out = 50))

pheatmap(TF_matrix, cluster_rows = FALSE, cluster_cols = FALSE, fontsize_row = 16,
         fontsize_col = 11, breaks = breaks_TF,
         color = colorRampPalette(c("blue", "white", "red"))(length(breaks_TF) - 1),
         cellwidth = 11, cellheight = 11, show_colnames = FALSE, gaps_row = c(1:35))
```

PHENYLPROPANOID PATHWAY PEROXIDASES

(1) Identify peroxidases relevant to lignin polymerisation

```r
Per_ID <- c("104450831", "104415764", "104434128", "104434134", "104421005",
                "104425008", "104422800", "104450451", "104449271")
```

(2) Extract relevant ENTREZ IDs from differentially expressed gene set

```r
Per <- gene_symbol %>%
  filter(grepl(paste(Per_ID, collapse = "|"), qseqid, ignore.case = TRUE),
         abs(log2FoldChange) >= 1.0, padj <= 0.05)
```

(3) Replace TAIR ID, with gene symbol

```r
Per$external_gene_name <- stri_replace_all_regex(Per$external_gene_name,
                                                 pattern=c("AT5G51890", "AT2G22420",
                                                           "AT1G71695", "PER64"),
                                                 replacement=c("PRX66", "PRX17",
                                                               "PRX12", "PRX64"),
                                                 vectorize=FALSE)
```

(4) Arrange based on Per_ID

```r
Per <- Per[order(match(Per$qseqid, Per_ID)), ]
Per <- rename(Per, p.adj = padj)
```

(5) Merge Gene ID and Symbol

```r
Per$ID_symbol <- paste(Per$qseqid, "(", Per$external_gene_name, ")", sep="")
```

(6) Plot data

```r
Per_order <- Per$ID_symbol

ggplot(Per, aes(x = log2FoldChange, y = factor(ID_symbol, levels = Per_order),
                fill = p.adj)) +
  geom_bar(stat = "identity", orientation = "y") +
  scale_fill_gradientn(colours=c("#b3eebe", "#46bac2", "#371ea3")) +
  labs(x = expression("Log"[2]~"fold change"),
       y = "Peroxidase",
       fill = "p.adj") + theme_minimal() +
  geom_errorbar(aes(x = log2FoldChange,
                    xmin = log2FoldChange - lfcSE, xmax = log2FoldChange + lfcSE),
                width = 0.4, colour = "black", alpha = 0.9, size = 0.02) +
  theme(panel.grid.major.x = element_line(color = "grey90", size = 0.5),
        panel.grid.major.y = element_line(color = "grey90", size = 0.5)) +
  theme(plot.margin = unit(c(0.5, 0.5, 0.5, 0.5), "lines")) +
  theme(axis.title.y = element_text(size = 22), axis.text.y = element_text(size = 22),
        axis.title.x = element_text(size = 22), axis.text.x = element_text(size = 22)) +
  theme( legend.text = element_text(size = 18), legend.title = element_text(size = 12),
         legend.key.size = unit(1.5, "lines")) +
  scale_x_continuous(breaks = seq(-2, 6, by = 2))
```

CREATING A CUSTOM MAPPING FILE FOR MAPMAN

(1) Load packages

```r
library("dplyr")
library("tidyverse")
library("stringi")
```

(2) Read in the MapMan arabidopsis thaliana mapping file

```r
mapping_file <- read.csv("./Data/input/Ath_AGI_ISOFORM_MODEL_TAIR10_Aug2012.csv", skip = 1)
colnames(mapping_file)[3] <- "sseqid"
mapping_file$sseqid <- sub("\\..*", "", sub("'", "", mapping_file$sseqid))
```

(3) Read in the deseq_orth file

```r
deseq_orth <- read.csv("./Data/input/deseq_orth.csv", head = TRUE)
deseq_orth$sseqid <- tolower(deseq_orth$sseqid)
```

(4) Merge deseq_orth to mapping_file to create a custom mapping file for E. grandis based on ENTREZ IDs
For example, the TAIR ID will be replaced by its corresponding E. grandis ENTREZ ID.
Merge dataframes but keep all rows for both.

```r
custom_mapping_E_grandis <- merge(deseq_orth[, c("qseqid", "sseqid")], mapping_file,
                                  by = "sseqid", all.y = TRUE)
custom_mapping_E_grandis$qseqid <- as.character(custom_mapping_E_grandis$qseqid)
```

(5) Fill empty rows in the qseqid column with TAIR IDs
Not all TAIR IDs had a corresponding ENTREZ ID, hence the blanks can be refilled with the original TAIR IDs.

```
custom_mapping_E_grandis <- custom_mapping_E_grandis %>% mutate(qseqid = coalesce(qseqid, sseqid))
```

(6) The original file had two single quotation marks ('') for the blank spaces and surrounding the gene IDs ('LOC123')

```
custom_mapping_E_grandis$qseqid <- ifelse(grepl("'", custom_mapping_E_grandis$qseqid),
                                          paste0("'", custom_mapping_E_grandis$qseqid),
                                          paste0("'", custom_mapping_E_grandis$qseqid, "'"))
```

(7) Reorder custom mapping file

```
custom_mapping_E_grandis <- custom_mapping_E_grandis[, -1]
colnames(custom_mapping_E_grandis)[1] <- "IDENTIFIER"
custom_mapping_E_grandis <- custom_mapping_E_grandis[, c(2, 3, 1, 4, 5)]
custom_mapping_E_grandis <- custom_mapping_E_grandis[order(custom_mapping_E_grandis[, 1]), ]
```

(8) Removing duplicate IDs
A single TAIR ID can have multiple matching ENTREZ IDs.
But since the ENTREZ IDs are unique, these wont be captured as duplicates in the mapping file.

```
check_duplicates <- custom_mapping_E_grandis[duplicated(custom_mapping_E_grandis$IDENTIFIER)
                                             | duplicated(custom_mapping_E_grandis$IDENTIFIER,
                                                          fromLast=TRUE), ]

custom_mapping_E_grandis <- distinct(custom_mapping_E_grandis)

length(unique(custom_mapping_E_grandis$IDENTIFIER))
```

(9) Write new custom mapping file as a tab delimited text file

```
write.table(custom_mapping_E_grandis, file = "./Data/output/custom_mapping_E_grandis.txt",
            sep = "\t", na = "", quote = FALSE, row.names = FALSE)
```
