# Supplementary material for: Xylem cell size regulation is a key adaptive response to water deficit in Eucalyptus grandis
Source: Tree Physiol. 2024 Jun 18;44(7):tpae068. doi: 10.1093/treephys/tpae068 (PMC11247191; doi:10.1093/treephys/tpae068)
Supplement: Method_S1_pilot_tpae068 [file method_s1_pilot_tpae068.pdf]

Method S1: *Eucalyptus grandis* pilot study to assess different watering regimes

1. Experimental design:

Four watering regimes (Table M1) were selected to determine what effect each would have on the physiological responses in *E. grandis*. Each watering regime comprised of six plants of approximately 45-50 cm in height that were placed into a growth chamber according to a randomized Latin square design (n = 6). The temperature was set to 25 degrees Celsius, with a day length of 16 h, and a dark period of 8 h. The soil composition consisted of palm peat and filter sand at a ratio of 1:1 (V/V), with 2 g of controlled-release fertilizer, contained within a 1.5 l bag. Numerous physiological parameters such as the pre-dawn leaf water potential, stomatal conductance (g_{sw}), transpiration and stem diameter were measured and served as a proxy to determine the physiological stress response in these plants.

Table M1.1. Water content administered to *Eucalyptus grandis* plants.

Percent field capacity	Water content	Actual water administered
15%	62.25 ml	60 ml
30%	124.5 ml	125 ml
45%	186.75 ml	180 ml
100%	415 ml	415 ml

2. Pre-dawn leaf water potential:

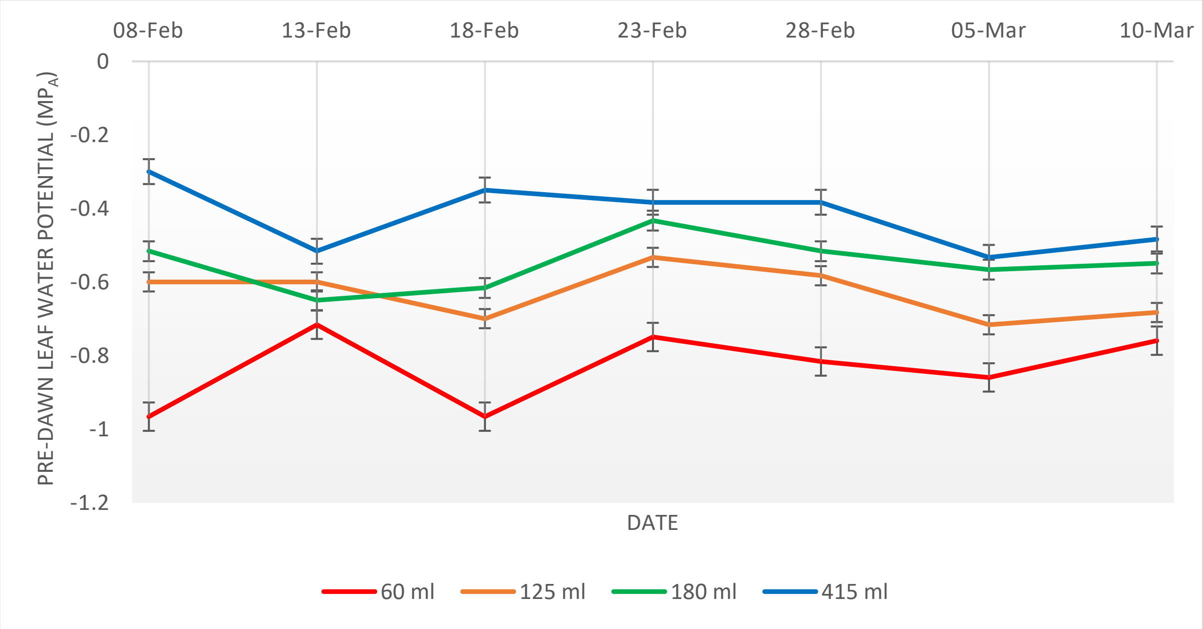

Figure M1.1. Pre-dawn leaf water potential of *Eucalyptus grandis* subject to different watering regimes over 30 days.

3. **Stomatal conductance:**

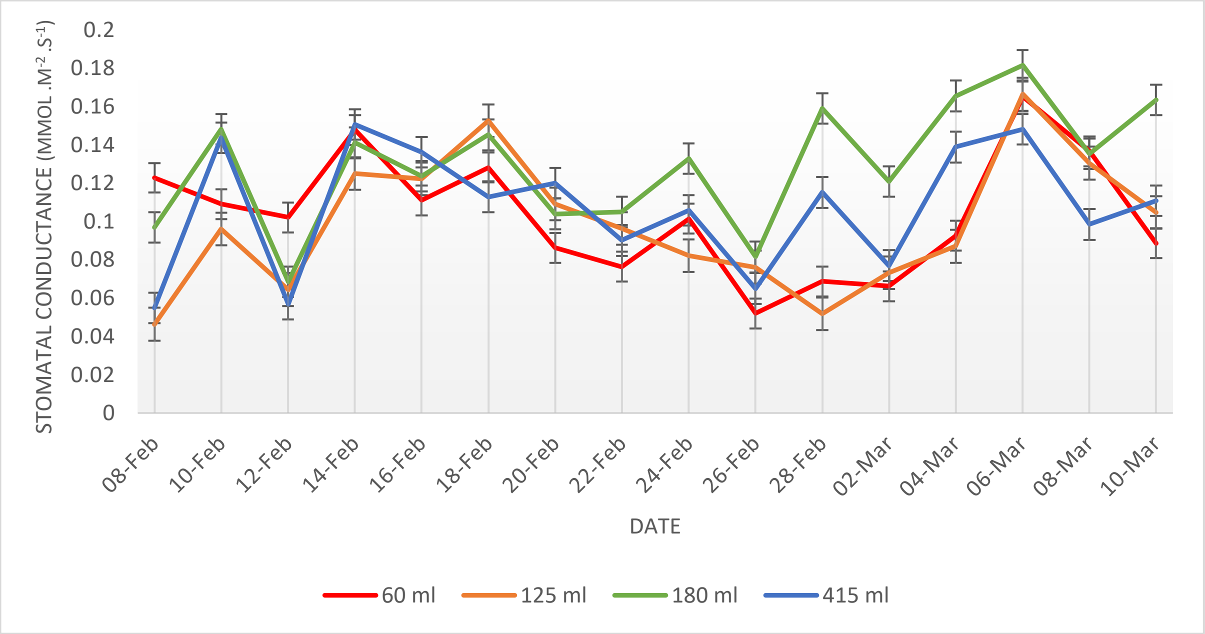

Figure M1.2. The stomatal conductance of *Eucalyptus grandis* subject to different watering regimes over 30 days.

4. **Transpiration rate:**

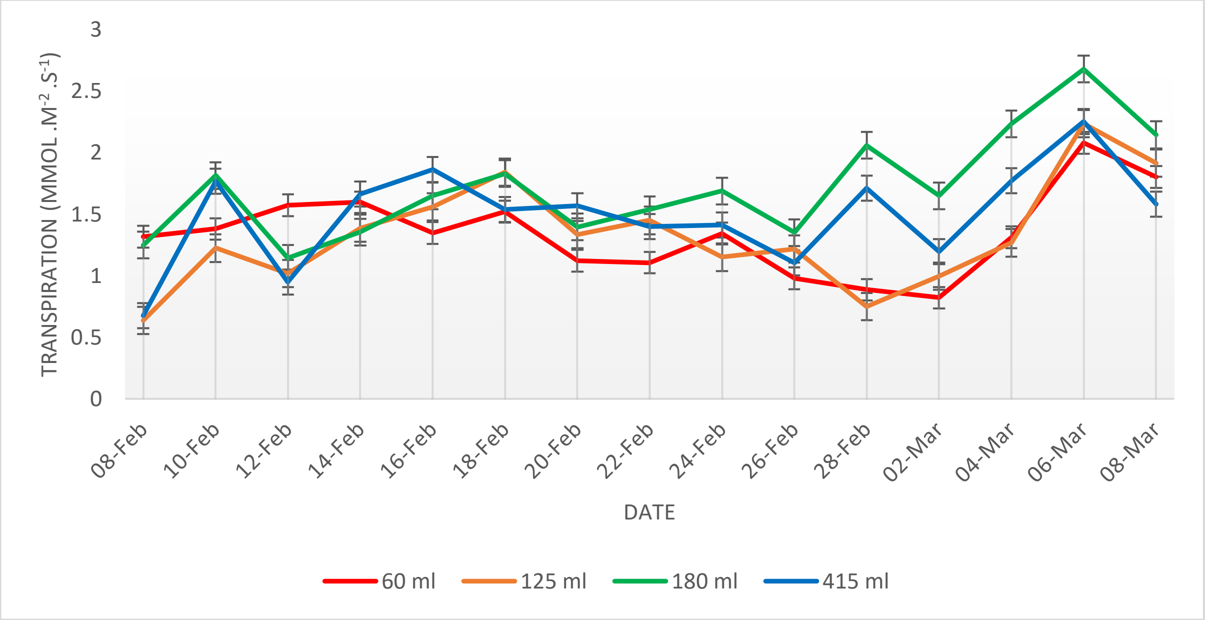

Figure M1.3. The transpiration rate of *Eucalyptus grandis* subject to different watering regimes over 30 days.

5. **Relative stem diameter:**

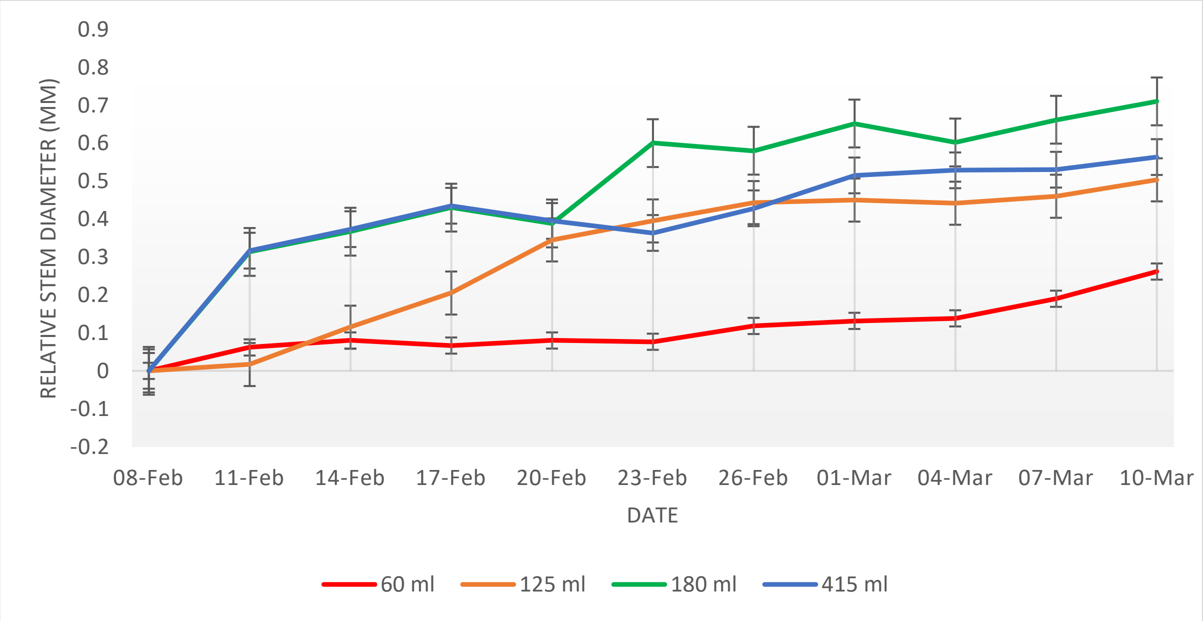

Figure M1.4. The relative change in stem diameter of *Eucalyptus grandis* subject to different watering regimes over 30 days.

6. **Discussion:**

From these results, it is evident that 60 ml of water daily leads to the lowest pre-dawn leaf water potential, indicating that the water column is under the highest tension for this treatment. The 180 ml and 415 ml treatments demonstrate higher (i.e. less negative) pre-dawn leaf water potentials, indicating that these plants have an overall higher water status, and are consequently experiencing less water stress. Considering the stomatal conductance and transpiration, it seems that the 180ml treatment produces the strongest response. This elevated stomatal conductance and transpiration has translated into the highest stem diameter growth in the plants receiving 180 ml of water. Although drought-stressed, the plants receiving 60 ml of water still grow steadily over the experimental period.

7. **Conclusion:**

Based on these findings, it seems that the 60 ml and 180 ml watering regimes provide the strongest physiological contrasts that may likely translate into anatomical differences in the wood anatomy and changes in gene expression in the transcriptome. These conditions will thus be selected as the droughted (60 ml) and control (180 ml) conditions for further experimentation.
