# Supplementary material for: Xylem cell size regulation is a key adaptive response to water deficit in Eucalyptus grandis
Source: Tree Physiol. 2024 Jun 18;44(7):tpae068. doi: 10.1093/treephys/tpae068 (PMC11247191; doi:10.1093/treephys/tpae068)
Supplement: Method_S2_ctab_tpae068 [file method_s2_ctab_tpae068.pdf]

Method S2: RNA extraction protocol

Table M2.1. General reagents and concentrations required for *Eucalyptus grandis* RNA extraction.

Reagents	Concentration in buffer	Stock Solution (100 ml)
CTAB	2%	2 g
PVP (Mw = 29)	2%	2 g
1M Tris-HCL (pH 8, use HCL)	100 mM	10 ml
0.5M EDTA	25 mM	5 ml
5M NaCl	2 M	40 ml
RNAse free water	-	45 ml

*Dissolve CTAB in water prior to adding the other reagents.
*Autoclave and add 2% (v/v) β-mercaptoethanol directly before use.

Prepare:

- 8 M Lithium Chloride (LiCl)
- Nuclease-free water
- Nuclease free 1.5ml / 2ml centrifuge tubes
- Chloroform: isoamyl-alcohol (24:1)
- 70% ethanol

Protocol:

1. Using a sharp minora blade or scalpel, remove the outer phloem/bark layer and take a 5 cm long cambial scraping of the stem, in the longitudinal direction. Refrain from scraping too deep into the stem, approximately 1-2 mm is sufficient. Repeat around the stem until the required amount of material is obtained.
2. Grind the stem material with liquid nitrogen, into a fine powder, and transfer 250-300 mg of powder into a 1.5 ml centrifuge tube.
3. Add 1 ml of RNA extraction buffer (containing 2% β-mercaptoethanol) to the ground material, and vortex / mix vigorously for 30 seconds.

4. Incubate the mixture of plant material and buffer at 65 degrees Celsius for 15-30 minutes, whilst vortexing / mixing every 5 minutes.
5. Centrifuge for 15 minutes at 16 000 *x*g at room temperature and transfer the supernatant into a fresh 1.5 ml tube.
6. Pipette 1 volume of chloroform/isoamyl alcohol to the supernatant, and vortex for 30 seconds.
7. Centrifuge for 15 minutes at 16 000 *x*g at 4 degrees Celsius and transfer the supernatant into a new tube.
8. Repeat steps 6 and 7. Which is the chloroform / isoamyl extraction.
9. Transfer the supernatant into a new 1.5 ml centrifuge tube and add LiCl to yield a final concentration of 2 M and incubate overnight or for 12 hours at 4 degrees Celsius to precipitate the RNA.
10. Centrifuge for 60 minutes at 4 degrees Celsius at 16 000 *x*g.
11. Discard the supernatant and wash the RNA pellet with 70-100% ethanol, remove the ethanol with a pipette.
12. Centrifuge for 20 minutes at 16 000 *x*g at 4 degrees Celsius and remove the residual ethanol via pipette.
13. Leave the samples to air dry for 15 minutes in a laminar flow.
14. Resuspend in 30 µl of nuclease-free de-ionized water.
15. Check RNA concentration and quality via nanodrop.
16. Load RNA onto gel to assess the integrity, and to determine if gDNA contamination is present.
17. Store at -80 degrees Celsius for later use.

Reference list:

White EJ, Venter M, Hiten NF, Burger JT (2008) Modified cetyltrimethylammonium

bromide method improves robustness and versatility: The benchmark for plant RNA extraction. Biotechnol J 3:1424–1428.

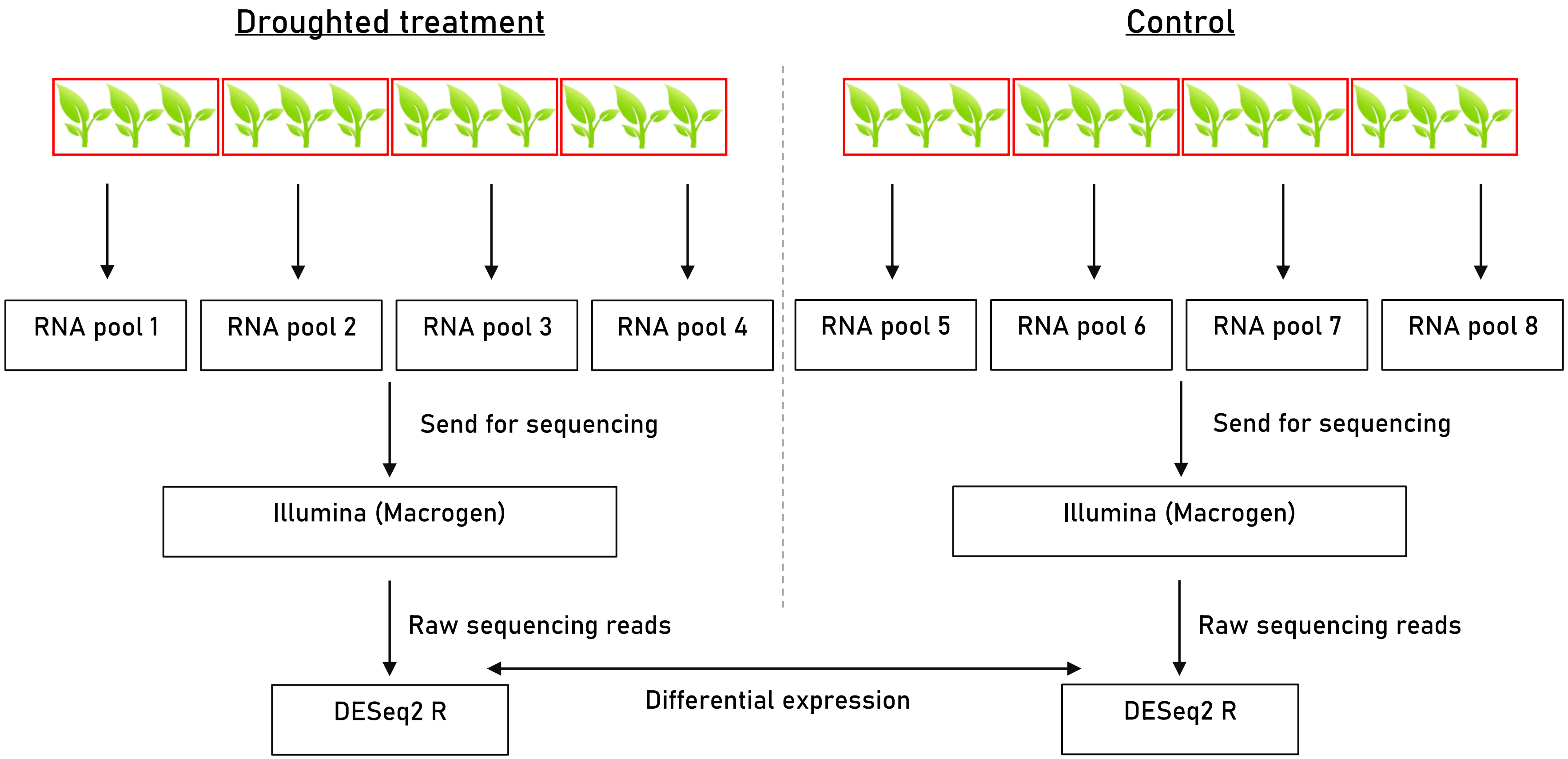

Figure M2.1. Illustration of the outline followed in order to generate RNA pools for RNA sequencing. In total, four RNA pools consisting of 12 plants were generated for both the control and droughted treatments (*n* = 4). These were sent to Macrogen for Illumina paired end sequencing. The raw sequencing data received from Macrogen was used for downstream differential expression analysis.
