# Supplementary material for: Xylem cell size regulation is a key adaptive response to water deficit in Eucalyptus grandis
Source: Tree Physiol. 2024 Jun 18;44(7):tpae068. doi: 10.1093/treephys/tpae068 (PMC11247191; doi:10.1093/treephys/tpae068)
Supplement: Method_S3_qc_tpae068 [file method_s3_qc_tpae068.pdf]

Method S3: RNA tissue enrichment

Experimental design:

1. This experiment is intended as a quality check to determine which tissues of the stem have been isolated during the RNA extractions. Once RNA was extracted, this was treated with DNase I to remove any potential genomic DNA contamination. The resulting RNA was reverse transcribed into cDNA for subsequent RT-PCR.
2. Primers specific to xylem, cambium and phloem tissues were designed to detect the presence of each tissue.
3. Considering the xylem-specific gene, a high-intensity band was observed in each sample.
4. For the cambium-specific gene, a lower-intensity band was present.
5. The phloem-specific gene was present as an exceptionally faint band on the gel.
6. The housekeeping gene was included to ensure that all RNA extractions and cDNA synthesis have been successful.

Table M3.1 RT-PCR primer design, for xylem, cambium, and phloem-specific gene amplification.

Tissue type	Accession number	Reference	Primers	Length (bp)	Gene
Xylem	Eucgr.J00938.1	Ko et al., 2012	Fp – ACAAAGTCAGCGTCTTCACC Rp – AGTCTTCACCTTCCCGACTT	131	FLAP11
Cambium	Eucgr.A02902	Zinkgraf et al., 2017	Fp – TGGCAAAGTTGATACAGAGC Rp – TCACTCTTCTCGTCGACTTG	106	PXY
Phloem	Eucgr.I01715.1	Nguyen et al., 2017	Fp – TATTCTGCCTCTTGTTGAGG Rp – ATAGCTCGTCCTGTGAGATG	113	DP3
All	-	de Almeida et al., 2010/2015	Not custom designed, retrieved from publication.	-	H2B

Table M3.2 RNA concentration and purity were measured on a NanoDrop Spectrophotometer.

Sample	Concentration (ng/µl)	A260/A280
C1	1112.2	2.14
C2	579.8	2.05
C3	748.4	2.00
C4	350.8	2.13
D1	667.1	2.00
D2	577.1	2.03
D3	635.7	2.03
D4	705.9	2.03

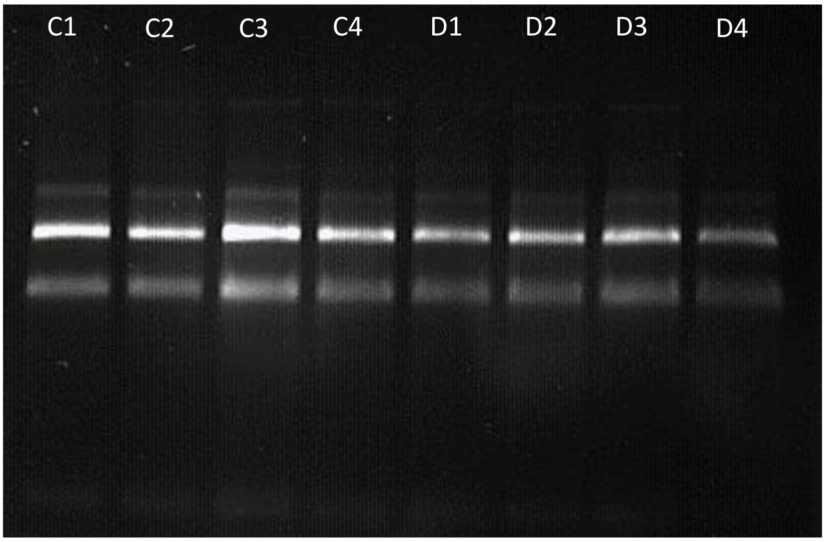

Figure M3.1 Agarose gel representing the RNA integrity for the eight *Eucalyptus grandis* pools. The 28s and 18s bands are clearly visible and show no noticeable signs of degradation or smearing on the gel.

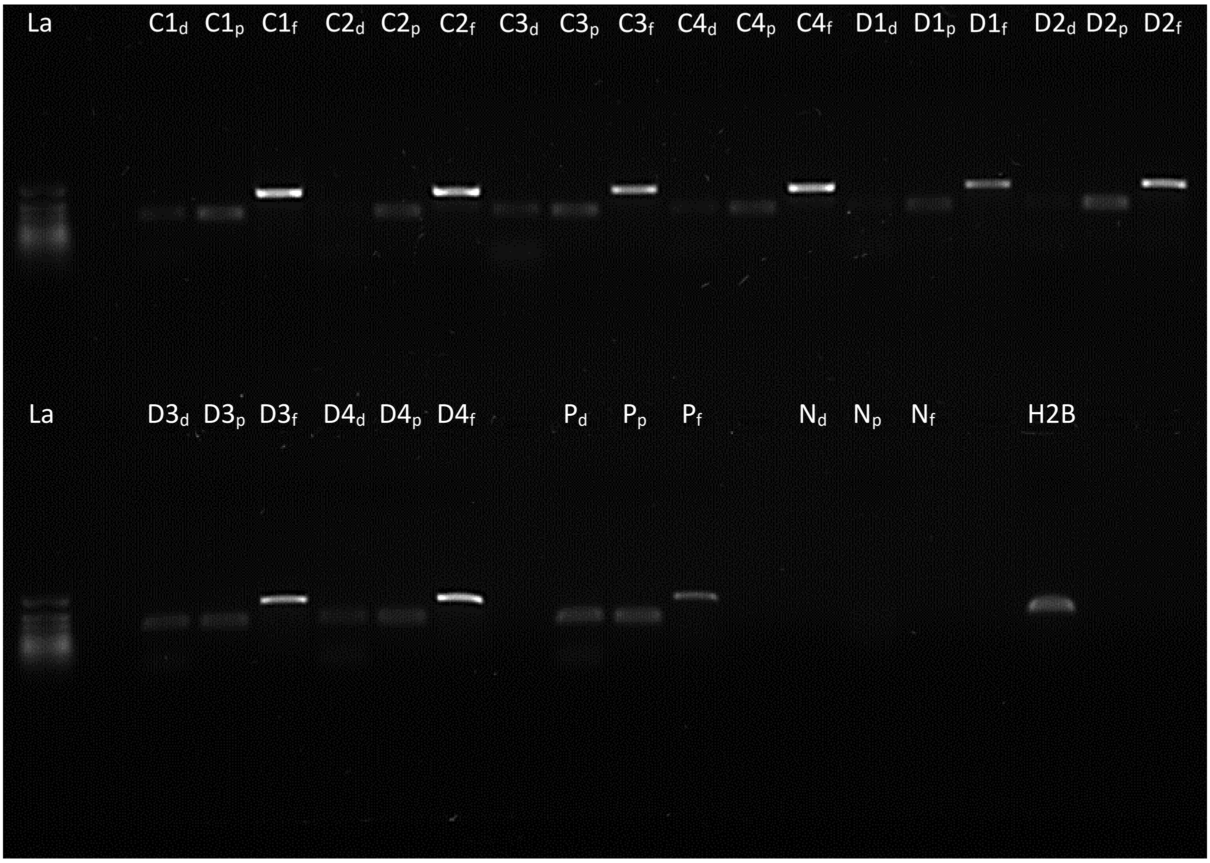

Figure M3.2 RT-PCR of the genes associated with the phloem, cambium, and xylem tissues for control (C) and droughted (D) treatments. The first lane represents the ladder (La). For the remainder of the lanes, the first letter (C/D) denotes the treatment, the number (1-4) denotes the RNA pool, and the final letters (d/p/f) denote phloem (d), cambium (p) or xylem (f) specific primers. Positive (P) and negative (N) controls for each primer set are included. H2B is the housekeeping gene. The annealing temperature used was 59 degrees Celsius.

Reference list:

de Almeida MR, de Bastiani D, Gaeta ML, de Araújo Mariath JE, de Costa F, Retallick J, Nolan L, Tai HH, Strömvik M V., Fett-Neto AG (2015) Comparative transcriptional analysis provides new insights into the molecular basis of adventitious rooting recalcitrance in *Eucalyptus*. Plant Sci 239:155–165.

Ko JH, Kim HT, Hwang I, Han KH (2012) Tissue-type-specific transcriptome analysis identifies developing xylem-specific promoters in poplar. Plant Biotechnol J 10:587–596.

Nguyen VP, Cho J-S, Lee J-H, Kim M-H, Choi Y-I, Park E-J, Kim W-C, Hwang S, Han K-H, Ko J-H (2017) Identification and functional analysis of a promoter sequence for phloem tissue specific gene expression from *Populus trichocarpa*. J Plant Biol 60:129–136.

Zhu A, Ibrahim JG, Love MI (2019) Heavy-Tailed prior distributions for sequence count data: Removing the noise and preserving large differences. Bioinformatics 35:2084–2092.
